# Co-targeting SRC overcomes resistance to BRAF inhibitors in colorectal cancer

**DOI:** 10.1038/s41416-025-03058-6

**Published:** 2025-06-06

**Authors:** Beatriz Rubio-Cuesta, Carlos Carretero-Puche, Patricia Llamas, Jacinto Sarmentero, Beatriz Gil-Calderon, Alberto Lens-Pardo, Beatriz Antón-Pascual, Eduardo Rubio-González, María Cámara-Jurado, Javier Salamanca, Daniel Rueda-Fernández, Marco Donatello Delcuratolo, Beatriz Soldevilla, Rocio Garcia-Carbonero

**Affiliations:** 1 Centro de Oncología Experimental. Grupo de Investigación en Tumores Gastrointestinales y Neuroendocrinos. Instituto de Investigación Sanitaria Hospital 12 de Octubre (imas12), Madrid, Spain; 2 Centro Nacional de Investigación Oncológica (CNIO), Madrid, Spain; 3 Oncology Department, Hospital Universitario 12 de Octubre, Madrid, Spain; 4 Department of General and Digestive Surgery, Hospital Universitario Doce de Octubre, Madrid, Spain; 5 Department of Pathology, Hospital Universitario 12 de Octubre, Madrid, Spain; 6 Hereditary Cancer Laboratory, 12 de Octubre University Hospital, i + 12 Research Institute, Madrid, Spain; 7 Facultad de Medicina, Universidad Complutense de Madrid (UCM), Madrid, Spain; 8 CIBERONC, Instituto de Salud Carlos III, Madrid, Spain

**Keywords:** Colon cancer, Translational research

## Abstract

**Background:**

*BRAF*^V600E^ mutations occur in ∼10% of colorectal cancer (CRC) patients, leading to poor prognosis. Although BRAF-targeted therapy is ineffective in CRC, adding EGFR inhibitors (EGFRi) improves efficacy, yet patient survival remains suboptimal. This study explores SRC as a key mediator of resistance to BRAF inhibitors (BRAFi) in preclinical BRAF^V600E^ CRC models, and its potential as a therapeutic target.

**Methods:**

We studied SRC using BRAF-mutated and wild-type CRC cell lines with CRISPR/Cas9 knockouts and lentiviral overexpression. We tested SRC, BRAF, EGFR, and JNK targeting drugs, assessing protein expression, cell viability, proliferation, migration, apoptosis, and cell cycle. CRC cell line-derived xenograft (CDX) and patient-derived xenograft (PDX) models were established for in vivo studies.

**Results:**

SRC regulates proliferation, clonogenicity, migration and mediates BRAFi resistance in BRAF^V600E^ CRC, regardless of microsatellite instability. Depletion or inhibition of SRC sensitized cells to BRAFi. Combined SRC and BRAF inhibition demonstrated a synergistic antitumor effect, reducing cell viability and inducing apoptosis and cell cycle arrest in cell lines and PDXs. The JNK/c-Jun pathway contributes to adaptive resistance, and its inhibition enhances the effects of dual SRC and BRAF inhibition.

**Conclusions:**

These findings identify new therapeutic targets for clinical trials, potentially improving outcomes for this high-risk CRC subgroup.

## Background

Colorectal cancer (CRC) is one of the leading causes of cancer-related death worldwide [1]. *BRAF* mutations, most commonly V600E, are present in ∼10% of CRC patients and are associated with a particularly poor prognosis [2, 3].

BRAF is a serine/threonine protein kinase that plays a critical role in the mitogen-activated protein kinase (MAPK) pathway, which regulates various essential cellular processes such as cell growth, proliferation, differentiation, and survival. Activating mutations of the *BRAF* oncogene (*BRAF*^V600E^) lead to constitutive activation of this pathway and contributes to cancer progression [3, 4]. In contrast with other BRAF^V600E^ tumors (i.e. melanoma), BRAF^V600E^-targeted therapy (like vemurafenib) has proven largely ineffective in CRC, with response rates <5% as single agents [5, 6]. Several mechanisms of innate resistance to BRAF inhibitors (BRAFi) in CRC have been identified, including a rapid EGFR feedback activation [7–9] or MAPK-pathway re-activation through multiple mechanisms [10–13]. Consequently, single targeted therapies are not effective due to the complex cross-talk between different receptor tyrosine kinase (RTK) downstream signaling cascades, suggesting that combination therapies with other targeted drugs may overcome this primary resistance to BRAF inhibition.

Based on this premises, vertical inhibition of the MAPK pathway at different points has been explored in multiple studies, assessing different combinations of BRAFi with EGFR and/or MEK inhibitors, as well as dual inhibition of the MAPK and PI3K/mTOR pathways [14–16]. More specifically, the phase III BEACON trial led to the approval of encorafenib (BRAFi) and cetuximab (EGRFi) for the treatment of pretreated metastatic BRAF^V600E^ CRC patients, as it demonstrated a significant survival improvement over the standard-of-care (irinotecan-based chemotherapy and EGFRi) (9.3 vs 5.9 months, HR 0.61, *P* < 0.001) [17, 18]. Despite these advances, however, patient survival is still rather poor and the development of more effective treatment strategies remains an unmet clinical need.

SRC kinase, a cytoplasmic non-receptor tyrosine kinase, has been implicated in the development and progression of cancer [19–22]. Higher expression and activity of SRC has been documented in primary colon tumors compared with benign polyps or non-transformed colonic mucosa, and increased activity is observed in progressive stages of CRC [23–26]. Moreover, aberrant SRC activation has been associated with a more aggressive phenotype and a worse prognosis in CRC patients [27, 28]. On the other hand, activation of SRC contributes to resistance to different cytotoxic drugs (i.e. platinum agents) in several malignancies [29–31] and preclinical studies suggest that inhibition of SRC may revert this resistance. Indeed, previous work from our group demonstrated that a high constitutive activation of SRC kinase was associated with resistance to oxaliplatin in CRC cell lines, and pharmacological inhibition of SRC sensitized CRC liver metastasis to oxaliplatin in orthotopically grown patient-derived xenograft (PDX) models [29]. SRC activation has been also implicated in innate or acquired resistance to targeted drugs, such as cetuximab in KRAS mutant CRC [32] or BRAFi in BRAF^V600E^ melanoma patients [33, 34], thus opening new avenues for potential drug manipulation that may eventually expand treatment options and improve the prognosis of these patients.

Based on these data, we hypothesized that SRC signaling may be largely responsible for drug resistance to targeted inhibition of the MAPK pathway in BRAF^V600E^ CRC. To this end, the objective of this study was to elucidate the role of SRC in BRAF^V600E^ CRC cell lines and to evaluate the antitumor activity of dasatinib (an orally bioavailable potent inhibitor of SRC (SRCi)) in combination with BRAFi (vemurafenib and encorafenib) using in vitro and in vivo models. This approach aimed to shed light on the intricate dynamics of BRAF^V600E^ CRC and pave the way for more effective therapeutic interventions.

## Material and methods

A detailed description of materials and methods is provided in the Supplemental Methods.

### Cell lines, cell culture and genetic alterations

Human colorectal cancer cell lines with *BRAF*^V600E^ mutations (HT29, SW1417, LS411N and RKO), and RAS and BRAF WT (DIFI) were obtained from American Type Culture Collection and Sigma-Aldrich. 293T and WiDr BRAF^V600E^ CRC cells were kindly provided by Dr. Luis Paz-Ares (Hospital 12 de Octubre, H12O-Centro Nacional de Investigaciones Oncológicas, CNIO, Madrid, Spain) and Dr. Jesús García-Foncillas (IIS- Fundación Jiménez Diaz, Hospital Fundación Jiménez Díaz, Madrid, Spain), respectively. See Supplementary Material and Methods for more details.

### Knock-out and overexpression of SRC in human colorectal cancer cell lines

Expression of SRC was modified in the human colorectal cancer cell lines by CRISPR/Cas9 (Knock-out (KO)) and pLenti-C-mGFP-P2A-Puro (overexpression) systems (Supplementary Material and Methods section). C-JUN overexpression was carried out by transient transfections of CRC cells with c-JUN plasmid (NM_002228) Human Tagged ORF Clone (Origene) and the respective control (pCMV6-AC-GFP Mammalian Expression Vector (PS100010)) (Origene) according to the manufacturer’s instructions.

### Drugs

Vemurafenib (PLX4032, BRAFi) and dasatinib (Sprycel®, Bristol-Myers Squibb, SRCi) were purchased from Selleckchem (Deltaclon, Madrid, Spain). Encorafenib (LGX818, BRAFi) was purchased from MedChemExpress (Eurodiagnostico, S.L. Madrid, Spain). Cetuximab (anti-EGFR) was kindly provided by the Pharmacy Department of Hospital Universitario 12 de Octubre. JNK inhibitor (JKNi) (SP600125, S5567) was purchased from Merck (Sigma-Aldrich). See drug specificity in Supplementary Table 1. All drugs, except for cetuximab, were dissolved in dimethyl sulfoxide (DMSO) for in vitro experiments. Control condition (vehicle) was treated with DMSO at concentrations lower than 0.1% (v/v).

For in vivo experiments, dasatinib and vemurafenib were dissolved in 4% DMSO, 20% PEG300, 5% Tween80 and 61% double-distilled H_2_O. Dasatinib and encorafenib were dissolved in 5% DMSO, 40% PEG300, 5% Tween80 and 50% saline. Cetuximab was used as an aqueous solution at a concentration of 5 mg/ml.

### Western blotting (WB)

Cells and frozen tumor tissue from PDX models were lysed in RIPA lysis buffer (Merck) with protease and phosphatase inhibitors (Merck). Protein concentration was calculated using the standard Pierce BCA Protein Assay (Fisher Scientific). Equal amounts of protein were separated on SDS-PAGE gels (10–12%), following which samples were blotted onto 0.2 µM pore size nitrocellulose transfer membranes (Fisher Scientific). Membranes were blocked with BSA 10% in TBS-Tween20 for 1 h and incubated with the appropriated primary antibodies overnight at 4 °C. A list of antibodies used is provided in Supplementary Table 2. Target bands were detected by incubating the membranes with ECL substrate (Amersham, DD Biolab) and then visualizing them on a Bio-Rad ChemiDoc MP imaging system. Images with protein bands were quantified using ImageLab software 6.1.

### Phospho-kinase array

HT29 cells were analyzed with a Human Phospho-Kinase Array (ARY003B, R&D System) after treatment with vemurafenib (0.4 µM) and/or dasatinib (0.3 µM). This array specifically screens for relative levels of phosphorylation of 39 individual proteins involved in cellular proliferation and survival. After treatments, cell lysates were incubated with membranes for 12 h and a cocktail of biotinylated detection antibodies, streptavidin-horseradish peroxidase and chemiluminescent detection reagents, were added to detect the phosphorylated proteins. The relative expression of the specific phosphorylated protein was determined following quantification of the scanned images using image analysis software (Image Quant TL 8.2).

### Cell viability assays

Cells were seeded at 5 × 10^3^–1 × 10^4^ cells/well in 96-well plates for 24 h and subsequently treated with different drugs. Following incubation for 3 days, plates were rinsed with PBS, fixed with 0.5% glutaraldehyde in PBS and stained with 1% (w/v) crystal violet solution (Merck) for 30 min at room temperature (RT). Plates were then washed with tap water and dried at RT. Twenty-four hours later, crystal violet was eluted with 20% acetic acid and absorbance was measured at 595 nm with a Victor Nivo multimode plate reader (Perkin Elmer). Drug response curves were generated and accompanying IC50 values were estimated using GraphPad Prism 8 software (GraphPad Software).

### Synergy assay

To determine the combinatorial effect of the tested drugs, 5 × 10^3^–1 × 10^4^ cells were plated overnight followed by treatment with increasing doses of BRAFi or SRCi, individually and in combination, at the defined ratio in 96-well plates. After 72 h, cell viability was measured by crystal violet assay (as described above) and graphed using GraphPad Prism 8. The combination indexes were determined by the median dose-effect method (Chou-Talalay analysis [35]) using CompuSyn software (ComboSyn, Inc.). The nature of the combinatorial interaction is interpreted on the basis of Combination Index (CI) values: CI < 0.9, synergism; CI = 0.9–1.1, additivity; CI > 0.9, antagonism. Data were obtained from three independent experiments and shown as the mean ± standard deviation (SD).

### Proliferation assay

5 × 10^2^ cells/well were seeded in 96-well plates in triplicate. After 24 h, cells were supplemented with 100 µL of fresh medium containing the specific drug/combination of drugs or vehicle. Cell proliferation data were obtained for 0, 24, 48, 72, and 96 h of treatment. The number of viable cells was determined with the CellTiter-Glo® Luminescent Cell Viability Assay (Promega) following the manufacturer’s instructions, with absorbance read on a Victor Nivo multimode plate reader (Perkin Elmer).

### Migration assays

1–2 × 10^5^ cells/well were plated in 300 µL of serum-free medium in the upper chamber of 8.0 µm transwell plates (Corning). In the bottom well, 400 µL of medium supplemented with 10% FBS was added to act as a chemoattractant. Treated cells and controls were incubated at 37 °C for 48–72 h. Non-migrating cells in the top of the chamber were removed and the remaining cells were quantified with CellTiter-Glo® according to the manufacturer’s instructions. Drug concentrations can be found in Supplementary Table 3.

### Colony formation assay

For clonogenic assays, 3–5 × 10^2^ cells/well were plated on 12-well plates and 24 h later, cells were treated with the indicated drugs (Supplementary Table 3) for 72 h. After 14 days, plates were fixed with 0.5% glutaraldehyde in PBS and stained with crystal violet (0.5% w/v) as described above. Colonies were photographed, counted and analyzed by ImageJ software (version 1.53e).

### Flow cytometry

To assess apoptosis, 4.5–5 × 10^5^ cells were seeded into 6-well plates and treated with different drugs and concentrations (Supplementary Table 3) for 48 h. Cells were collected and stained with Annexin V FITC and 7-Amino-Actinomycin D (7-AAD) according to the manufacturer’s instructions in the detection kit (ANXVKF7-100T, Immunostep Salamanca, Spain),

Cell cycle assays were performed by seeding 4.5–5 × 10^5^ cells in 6-well plates and treating them with drugs at the indicated concentrations (Supplementary Table 3). 24 h later, cells were washed with PBS, fixed with pre-cooled 70% ethanol, and stored at −20 °C overnight. Fixed cells were then washed with PBS, subjected to RNase I treatment and stained with propidium iodide (PI, 1 mg/ml) for 30 min in the dark.

Results were acquired on The Becton Dickinson (BD) FACSCalibur Flow Cytometer and analyzed by FlowJo^TM^ software (Version 10.6.1, BD Biosciences).

### CRC cell line xenograft and PDX models

Cell line-derived xenograft (CDX) and PDX models were performed. For CDX models, 7.5 × 10^5^ HT29 CRC cells were subcutaneously injected in the right flanks of female athymic nude mice (Crl:NU(NCr)-*Foxn1*^*nu*^, 4–6 weeks old) (Charles River Laboratories, France). PDX models were established from fresh tumor samples derived from patients undergoing surgical resection of CRC at Hospital 12 de Octubre as previously described [29].Tumor pieces were engrafted into the right flanks of female athymic nude mice (Supplementary Fig. 1). Mouse experiment conditions and patients information are detailed in Supplementary Material and Methods.

### Histopathology and molecular analysis

Harvested tissues from all PDX models were fixed in 10% buffered formalin within 30 min of resection. After 24 h of tissue fixation, standard procedures were followed for further tissue processing (see Supplementary Material and Methods section).

For molecular characterization of the tumors, *KRAS*, *NRAS* and *BRAF* status was evaluated at the Pathology Department of Hospital 12 de Octubre, by performing a fully automated KRAS, NRAS or BRAF mutation test based on real-time PCR, Idylla KRAS, NRAS or BRAF, respectively - Mutation Test (Biocartis). MSI or deficient mismatch repair (dMMR) status was assessed by immunohistochemistry (IHC) and/or polymerase chain reaction (PCR) at the Molecular Pathology Department of Hospital Universitario 12 de Octubre (see Supplementary Material and Methods section).

### Statistics

Data are presented as mean ± SD or mean ± SEM (standard error of mean) of at least 3 independent experiments (unless otherwise noted). GraphPad Prism 8 was used to perform statistical analyses. To assess the normality of data distribution we performed the Shapiro–Wilk test. Data distribution was assumed to be homoscedastic, but this was not always formally tested. To evaluate differences between groups, two-sided unpaired Student’s *t* tests or Mann–Whitney U tests were performed. A *P*-value < 0.05 was considered statistically significant. The following symbols were used to denote the statistical significance of data: ns non-significant, *P < 0.05, **P < 0.01, ***P < 0.001 and ****P < 0.0001. Data collection and analysis were not performed with blinding to the conditions of the experiments. No data were excluded from analyses.

## Results

### SRC expression modulates oncogenic capacities in BRAF^V600E^ CRC

We first evaluated the basal expression levels of phosphorylated SRC (pSRC) in a set of five CRC cell lines with the *BRAF*^V600E^ mutation (HT29, SW1417, WiDr, LS411N and RKO) (Supplementary Fig. 2A). PSRC expression was similar among the different BRAF^V600E^ cell lines, except for LS411N, which displays the highest levels (Supplementary Fig. 2A).

To further explore the impact of SRC on BRAF^V600E^ CRC, we overexpressed or knocked out the *SRC* gene in our set of BRAF^V600E^ cell lines (Supplementary Fig. 2B). Overexpression of SRC significantly promoted cell proliferation and migration, as well as the ability of colony formation in the HT29 cell line, a well-established BRAF^V600E^ CRC model (Supplementary Fig. 3A, C, E). Similar results were found in clonogenic assays of the remaining CRC cell lines (Supplementary Fig. 4). Accordingly, *SRC* depletion led to a reduction of cell growth and migration in HT29 cells (Supplementary Fig. 3B, D, F), as well as in the number of colonies of all cell lines (Supplementary Fig. 4).

We then examined the role of SRC in BRAF^V600E^ CRC in vivo. To this aim, HT29 SRC KO or SRC overexpressing cells, as well as their mock controls, were injected into the right flank of nude mice (Supplementary Fig. 3G). Tumors derived from HT29 SRC OE were significantly larger than those derived from mock cells, while HT29 SRC KO showed smaller tumors. Of note, HT29 OE SRC tumors initially grow at a similar rate to CTRL up to day 55; thereafter, the OE tumors exhibit significantly faster growth, reaching a larger volume by the end of the study (Supplementary Fig. 3H, I). Altogether these findings demonstrate the crucial role of SRC modulating oncogenic capacities and highlight its relevance in the context of BRAF^V600E^ CRC.

### BRAF inhibition induces SRC activation in BRAF^V600E^ CRC

In contrast to BRAF^V600E^ melanomas, treatment with single-agent BRAFi has provided disappointing results in CRC [7–13]. Among them, SRC activation has been implicated in resistance to drugs targeting the MAPK pathway, including EGFRi in KRAS-mutated CRC or BRAFi in BRAF-mutated melanoma. This prompted us to explore the role of SRC in mechanisms of resistance to BRAFi in BRAF^V600E^ CRC preclinical models.

**Fig. 1:**
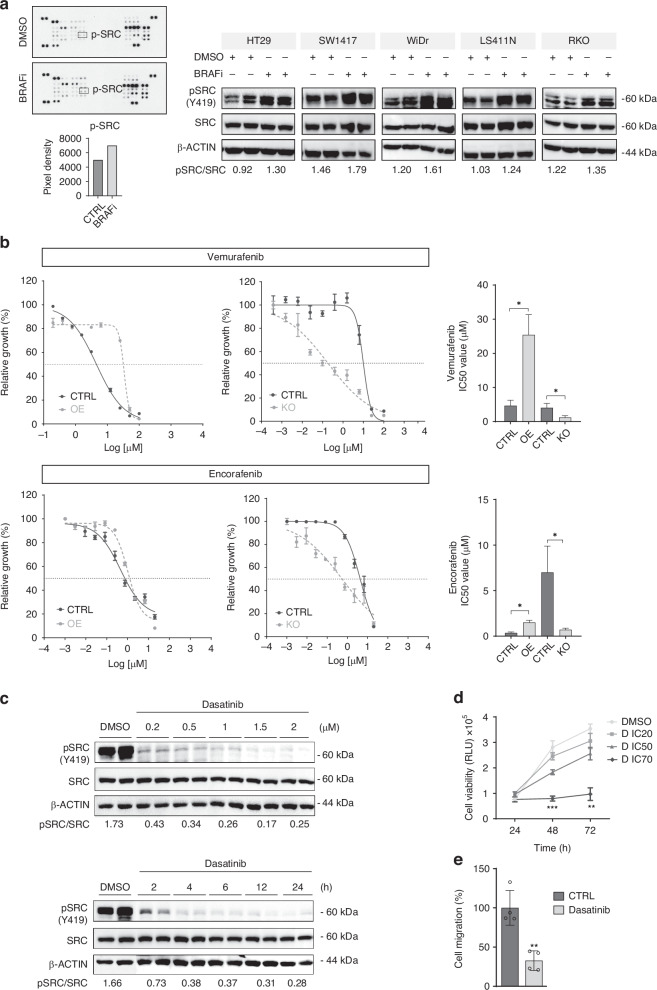
SRC is activated following BRAF inhibition in BRAFm CRC cell lines and efficiently inhibited by dasatinib. **a** On the left, proteome human phospho-kinase array of HT29 cell line following vemurafenib treatment (IC50 value, 24 h). The black boxes in the figure highlight pSRC (Y419) upregulation, and the pixel densitometry representation of this data is shown below. On the right, expression of SRC and pSRC in the panel of CRC BRAF^V600E^ cell lines treated with BRAFi (vemurafenib (HT29, SW1417 and WiDr) or encorafenib (LS411N and RKO)) at their IC50 values for 8 h. SRC activation is reflected by increased phosphorylation of the SRC activation site Y419 (pY419). β-actin used as a loading control. Molecular weight/size markers are indicated on the right (kDa). Images of WB are representative of 3 independent experiments. **b** Dose-response curves of HT29 cells (SRC OE and SRC KO cells) after treatment with vemurafenib or encorafenib for 72 h (left). On the right, representation of IC50 values of modified HT29 cells after treatment with vemurafenib or encorafenib for 72 h, compared to controls. Data represent the mean ± SEM of at least 3 biologically independent experiments. **c** Representative WB analysis of pSRC and SRC in HT29 cells after being treated at different concentrations (0.2–2 µM) for 24 h and with 1.2 µM dasatinib at the indicated time points (1–24 h), and their respective controls. β-actin used as a loading control. Data are representative of 3 independent experiments. Protein quantification is shown below as a pSRC/SRC ratio. **d** Dose-response assay of HT29 treated with dasatinib for 72 h, after treatment with the IC20 (0.06 µM), IC50 (1.20 µM) and IC70 (4.11 µM) doses (*n* = 3 replicates examined). **e** Transwell assay for cell migration in HT29 cells after being treated with dasatinib or control (DMSO) for 24 h. Transmigrated cells were quantified by cell titter. Error bars represent the mean ± SD of 4 biologically independent experiments. Significance was considered for *P < 0.05, **P < 0.01 and ***P < 0.001. D dasatinib, IC20 20% inhibitory concentration, IC50, half-maximal inhibitory concentration, IC70 70% inhibitory concentration, CTRL control, OE overexpression, KO knock-out.

First, IC50 values for both BRAFi (vemurafenib and encorafenib) were obtained for all CRC cell lines (Supplementary Fig. 5A). Interestingly, after treating BRAF^V600E^ cells with both BRAFi for 8 h, phosphorylation of SRC was upregulated, when compared to control conditions (Fig. 1a), indicating that SRC activation occurs as an adaptive response to BRAF inhibition.

To further test our hypothesis that resistance to BRAFi may be mediated through SRC as a common signal transduction node, we modulated the expression of SRC and assessed the response of BRAF^V600E^ CRC to specific BRAFi. HT29 SRC overexpressing cells were significantly more resistant to vemurafenib, while SRC depletion resulted in an increased sensitivity (Fig. 1b). Accordingly, IC50 values were up to five times higher in SRC overexpressing cells and four times lower in SRC KO cells when compared to respective controls. Similar data were obtained with another BRAFi, encorafenib (Fig. 1b), and with the entire panel of CRC cell lines assessed (WiDr, SW1417, RKO, LS411N) (Supplementary Fig. 5B). However, response to BRAFi was unaffected in RAS/BRAF WT CRC cell lines (DIFI) when the expression of SRC was modified (Supplementary Fig. 5C). Overall, these data support the implication of SRC in the resistance to BRAFi in BRAF^V600E^ CRC.

### Dual inhibition of SRC kinase and BRAF is synergistic in BRAF^V600E^ CRC

Based on the prior data, we aimed to explore if resistance to BRAFi would be reverted by targeting SRC, and proposed a novel treatment strategy for BRAF^V600E^ CRC based on dual inhibition of SRC (dasatinib) and BRAF (vemurafenib or encorafenib).

First, we evaluated the effect on SRC of dasatinib, a potent EMA- and FDA-approved SRC inhibitor (SRCi), in our BRAF^V600E^ in vitro model. We thus corroborated the activation of SRC was fully inhibited in all evaluated BRAF^V600E^ cell lines after treatment with their corresponding dasatinib IC50 values (Supplementary Fig. 6). More specifically, in HT29 cells, pSRC levels rapidly decreased after 2 h of dasatinib treatment, although the highest inhibition was obtained at 24 h. HT29 cells were also treated with increasing concentrations of dasatinib (from 0.2 to 2 µM) and we found that the inhibition of SRC activation occurred in a dose-dependent manner (Fig. 1c). Moreover, dasatinib treatment inhibited the proliferation and migration of HT29 cells in a concentration-dependent manner (Fig. 1d, e).

#### Cell viability

**Fig. 2:**
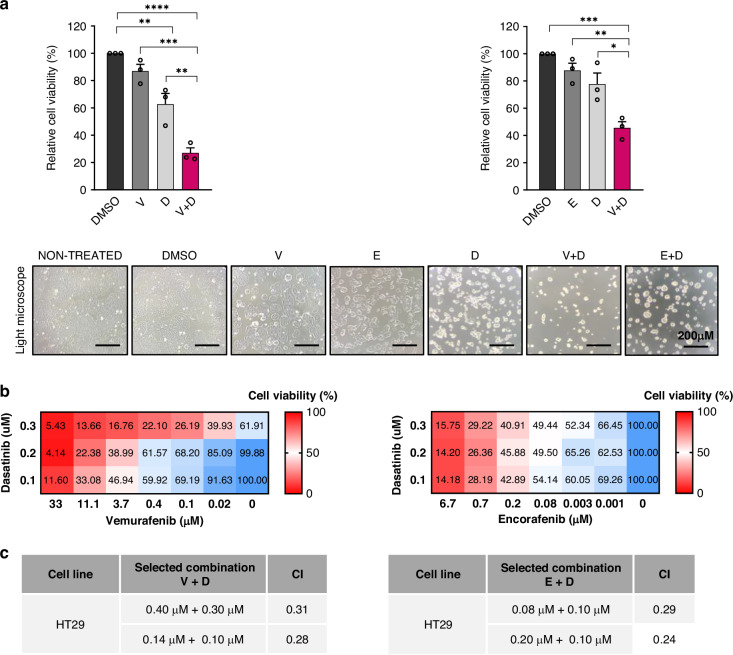
Synergistic interaction of SRCi and BRAFi in MSS BRAFm CRC. **a** Cell viability after a 72 h treatment with IC50 values of SRCi (dasatinib), BRAFi (vemurafenib and encorafenib) and the combination of both in HT29 cell line. Error bars represent the mean ± SEM of 3 biologically independent experiments. Below, representative images under light microscope of cell viability after 48 h of the indicated treatments. Scale bar, 200 µm. **b** Heatmaps of cell viability assays for the combination of increasing concentrations of the drugs after data normalization in 0–100 scale. 0 means strong cell killing. **c** Selected concentrations of BRAFi and SRCi with the CI value, obtained with compusyn software. CI < 0.9 means synergy. Significance was considered for *P < 0.05, **P < 0.01, ***P < 0.001, and ****P < 0.0001. V vemurafenib, E encorafenib, D dasatinib, CI combination index.

**Fig. 3:**
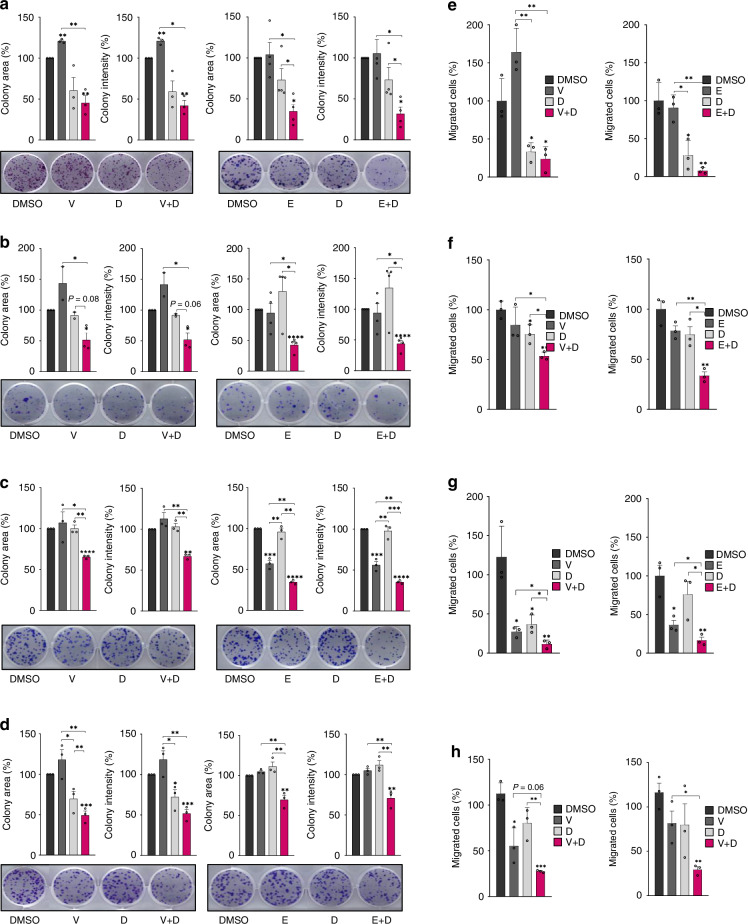
Dual SRC and BRAF inhibition leads to a reduction in cell clonogenicity and migration in BRAFm CRC cell lines. CFAs for HT29 (**a**), SW1417 (**b**), WiDr (**c**) and RKO (**d**) cell lines. Drug doses: V (0.14, 0.08, 0.08, 0.60 (μM)), E (0.08, 0.03, 0.05, 0.016 (μM)), D (0.10, 0.001, 0.003, 0.10 (μM)). Left graphs represent the quantification of the area and intensity of the colonies after treatment with SRCi (dasatinib) plus BRAFi (vemurafenib, left) or BRAFi (encorafenib, right), as well as their respective controls (vehicle, BRAFi and SRCi in monotherapy). Data represent the mean ± SEM of 3 biologically independent experiments. Below the graphs, representative images of the colonies after crystal violet staining. On the right, transwell assay for cell migration in HT29 (**e**), SW1417 (**f**), WiDr (**g**) and RKO (**h**) cell lines. Drug doses: V (0.40, 0.08, 0.08, 1.850 (μM)), E (0.08, 0.005, 0.008, 0.016 (μM)), D (0.10, 0.001, 0.003, 0.10 (μM)). Transmigrated cells were quantified by cell titter relative to control. Data represent the mean ± SEM of 3 biologically independent experiments. Asterisk above the bar indicates significance between that groups versus the control, square brackets indicate it for the pointed groups. Significance was considered for *P < 0.05, **P < 0.01, ***P < 0.001, and ****P < 0.0001. V vemurafenib, E encorafenib, D dasatinib.

We then explored the cytotoxic effect of dual SRC and BRAF inhibition and confirmed that combined treatment significantly reduced HT29 cell viability compared to single agent treatment (Fig. 2a). To further explore the drug interaction between SRCi (dasatinib) and BRAFi (vemurafenib or encorafenib), cells were treated with increasing concentrations of both drugs as per the Chou-Talalay algorithm [35]. A strong synergistic effect was found in HT29 cells after dual SRC and BRAF inhibition (Fig. 3b, c). Similar findings were obtained in the other BRAF^V600E^ CRC cell lines (SW1417, WiDr, LS411N and RKO) (Supplementary Fig. 7). These results suggested that SRCi and BRAFi mutually cooperate to induce cell death in BRAF^V600E^ CRC cell lines.

#### Colony formation and cellular migration

HT29, SW1417, WiDr and RKO cells treated with the combination of SRCi (dasatinib) and BRAFi (vemurafenib or encorafenib) exhibited significantly reduced colony formation (intensity and area of colonies) compared to single drug treatment or under control conditions (Fig. 3a–d). Additionally, SRCi plus BRAFi treatment also decreased the migration of cancer cells in a significant manner compared with monotherapy or under control conditions (Fig. 3e–h).

#### Apoptosis and cell cycle arrest

**Fig. 4:**
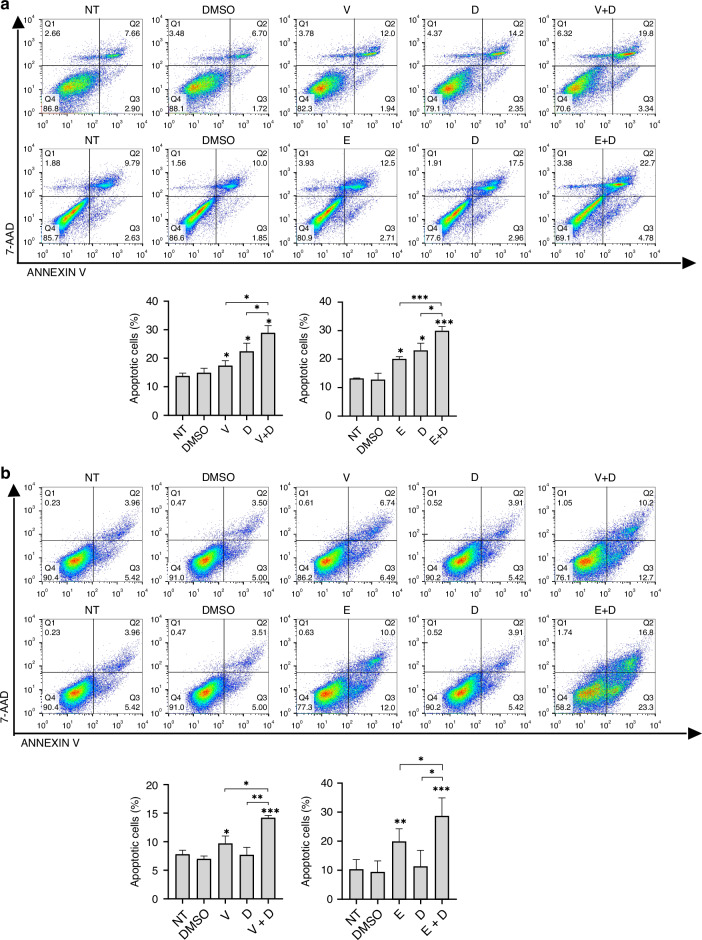

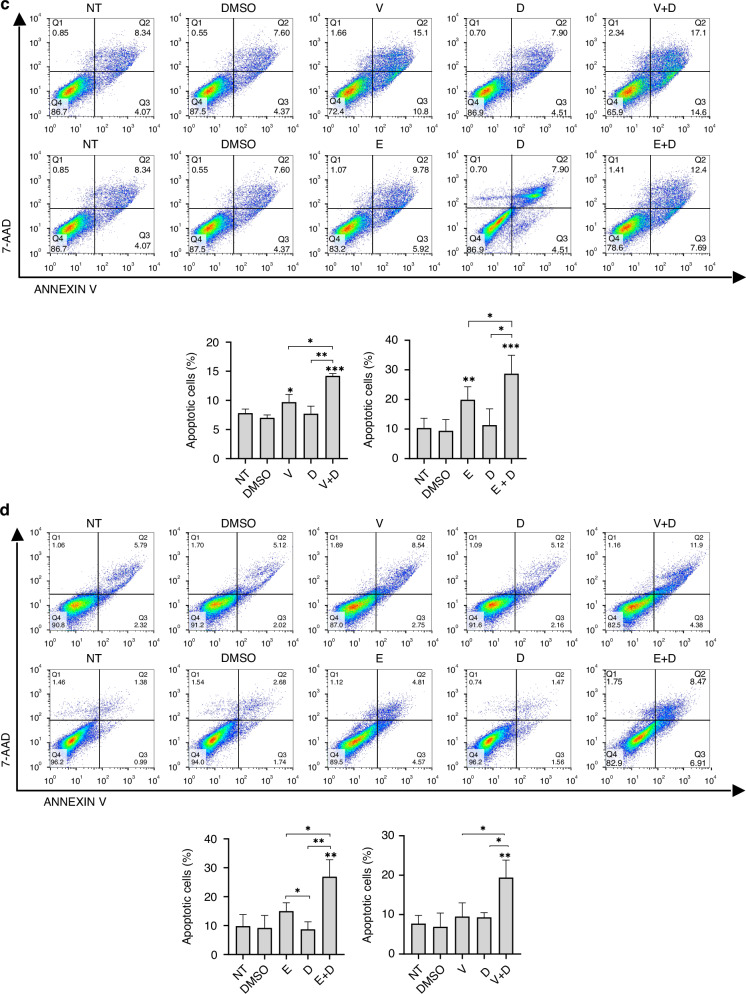

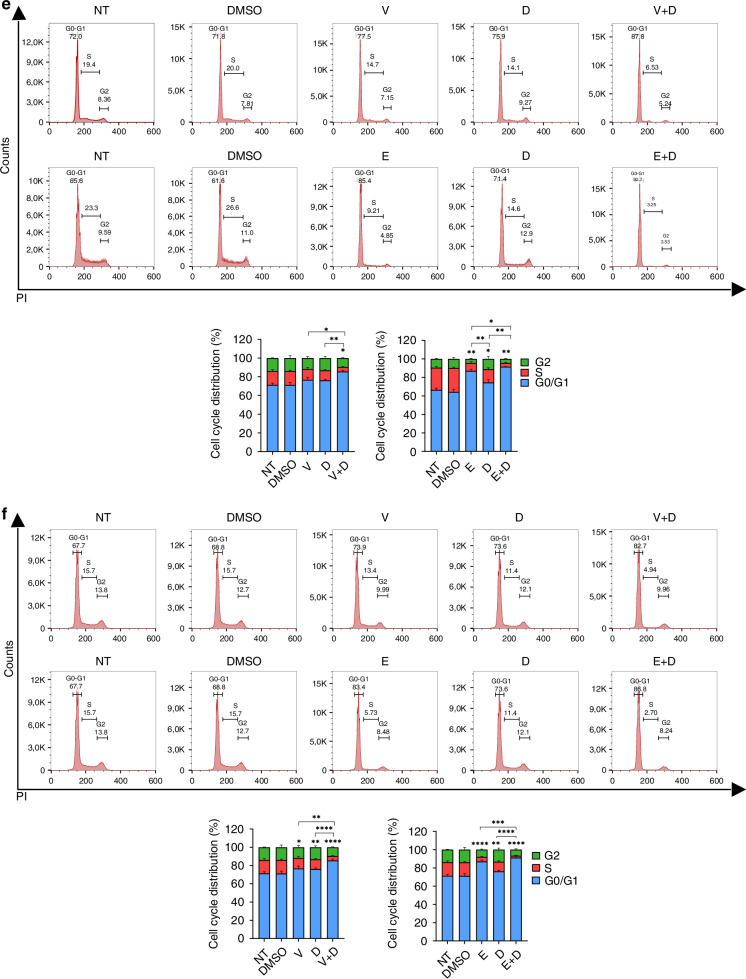

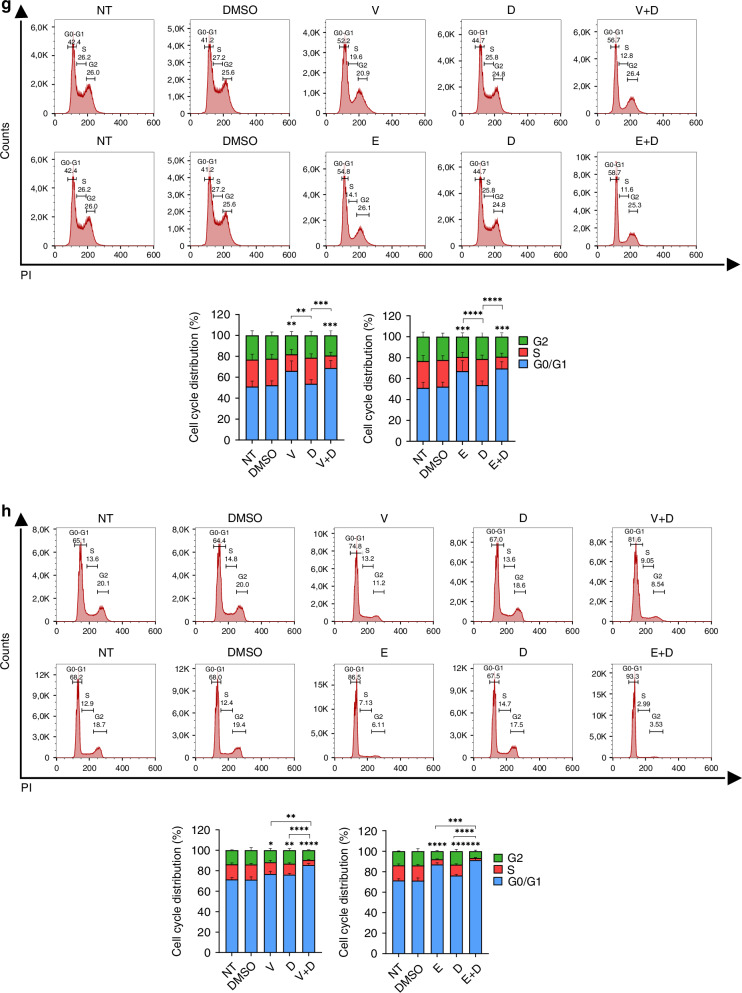
SRCi plus BRAFi promotes apoptosis and cell cycle arrest in BRAFm CRC cells. Representative apoptotic profiles of Annexin V-APC/7-AAD staining of HT29 (**a**), WiDr (**b**), LS411N (**c**) and RKO (**d**) cell lines after the indicated treatments V (0.40, 1.50, 0.35, 1.85 (μM)), E (0.08, 2.00, 0.001, 2.00 (μM)), D (0.10, 0.03, 0.10, 0.10 (μM))) for 48 h. Below, histogram representation showing apoptotic rates. Mean and SD of 5 independent experiments are shown. Significance was considered for *P < 0.05, **P < 0.01, ***P < 0.001. Representative images of cell cycle distribution of HT29 (**e**), WiDr (**f**), LS411N (**g**) and RKO (**h**) cell lines after the indicated treatments (V (0.40, 0.10, 2.10, 1.85 (μM)), E (0.08, 0.30, 0.37, 0.60 (μM)), D (0.10, 0.02, 0.10, 0.10 (μM))) of one representative replicate are shown. Below, a bar diagram representation shows the percentage of G0/G1, S and G2 cells in each condition, showing mean ± SD of at least 3 biologically independent experiments. Asterisk above the bar means significance between that groups versus the control (DMSO), square brackets indicate it for the pointed groups. Significance is shown for S phase, considered for * *P* < 0.05, ** *P* < 0.01, *** *P* < 0.001, and **** *P* < 0.0001. NT non-treated, PI Propidium iodide, NT non-treated, V vemurafenib, E encorafenib, D dasatinib.

To further elucidate the anti-proliferative mechanisms of dual SRC and BRAF inhibition, we used flow cytometry to analyze drug-induced apoptosis and cell cycle changes. BRAF^V600E^ CRC cells treated with SRCi (dasatinib) and BRAFi (vemurafenib or encorafenib) showed a higher ratio of apoptotic cells than those treated with monotherapy (Fig. 4a–d). Additionally, a significant reduction of the cell cycle phase S and an increase in the number of cells in the G0/G1 phase was observed in BRAF^V600E^ CRC cells after exposure to the combination treatments (Fig. 4e–h).

### c-JUN as a potential mediator of acquired resistance to BRAF inhibition in BRAF^V600E^ CRC

**Fig. 5:**
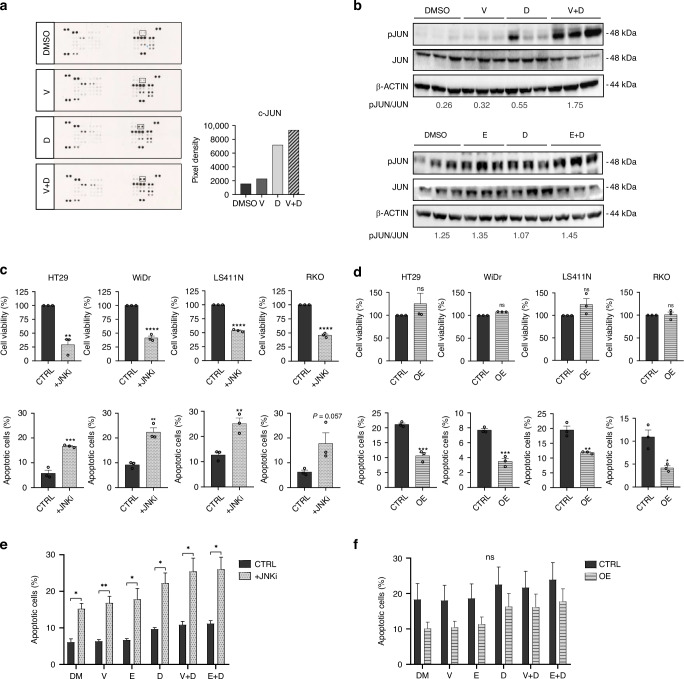
Role of c-JUN on cell survival in BRAFm CRC cell lines. **a** Scanned membranes of the proteome human phospho-kinase array revealed upregulation of c-JUN in HT29 cell line after dual inhibition of SRC and BRAF. On the right, graphical representation of the pixel density quantification of the membranes using scanned images from Image Quant TL software. **b** Representative WB analysis of phosphorylated and total JUN in HT29 cell line after the indicated treatments. Images of WB are representative of 3 independent experiments. β-actin was used as a loading control. Molecular weight/size markers are indicated on the right (kDa). pJUN/JUN ratio is shown below. **c** BRAFV600E CRC cell lines were treated with JNKi (SP600125) or with the vehicle (CTRL) for 24 h, and cell viability relative to control and apoptosis were measured by crystal violet or by Annexin V-APC/7-AAD staining by flow cytometry, respectively. Mean and SEM of 3 independent experiments are shown. **d** BRAFV600E CRC cell lines were transiently transfected with OE JUN plasmid or control plasmid (CTRL), and cell viability relative to control and apoptosis were measured by crystal violet or by Annexin V-APC/7-AAD staining by flow cytometry, respectively. Average and SEM of 3 independent experiments are shown. Flow cytometry analysis measured by Annexin V-APC/7-AAD staining of HT29 after treatment with JNKi (**e**) or after c-JUN overexpression (**f**); compared to control cells, and following the indicated treatments for 24 h (*n* = 5). Mean and SEM of 3 independent experiments are shown. Significance was considered for *P < 0.05, **P < 0.01 and ***P < 0.001. Ns non-significant, CTRL control, OE overexpression of c-JUN, JNKi JNK inhibitor (30 µM), DM DMSO, V vemurafenib, E encorafenib, D dasatinib.

To explore the molecular mechanisms triggered upon dual SRC and BRAF inhibition, we performed a proteome profile of human phospho-kinases in HT29 cells treated with dasatinib and/or vemurafenib. We observed that c-JUN phosphorylation was increased upon exposure to combined SRCi and BRAFi as compared to single agents or vehicle control (Fig. 5a). c-JUN activation after dual SRCi and BRAFi was confirmed by WB for both BRAFi (vemurafenib and encorafenib) (Fig. 5b).

As JUN may play a dual role in cell survival [36, 37], and to further evaluate its contribution in BRAF^V600E^ CRC cell lines, we transiently modulated its expression (Supplementary Fig. 8A). We used SP600125 inhibitor (S5567) owing to its ability to suppress the JNK/c-JUN pathway (Suplementary Table 1). A significant decreased cell growth and remarkable increased apoptotic rate was observed when BRAF^V600E^ CRC cell lines were treated with the JNK/c-JUN pathway inhibitor, as compared to the control (Fig. 5c). In contrast, overexpression of c-JUN showed no effect or a slight trend to higher cell proliferation and a significant decrease in apoptosis in all BRAF^V600E^ CRC cell lines tested (Fig. 5d). We then investigated the role of c-JUN in modulating apoptosis induced by SRC and/or BRAF-targeted drugs in HT29 cells. In line with the previous results (Fig. 5c, d), cytometry analysis showed that inhibition of c-JUN led to a significant increase of apoptotic rates when compared to the control (Fig. 5e), both after single or dual SRC and/or BRAF inhibition. On the contrary, c-JUN overexpression led to decreased apoptosis in these cell lines upon single or dual inhibition of SRC and/or BRAF (Fig. 5f). Similar results were obtained in the remaining set of BRAF^V600E^ CRC cell lines (Supplementary Fig. 8B). Globally, these data indicate that c-JUN plays an anti-apoptotic role and may be a potential mediator of acquired resistance to BRAF inhibition in BRAF^V600E^ CRC.

### Dual SRC kinase and BRAF inhibition confirms relevant antitumor efficacy in BRAF^V600E^ CRC in vivo models

**Fig. 6:**
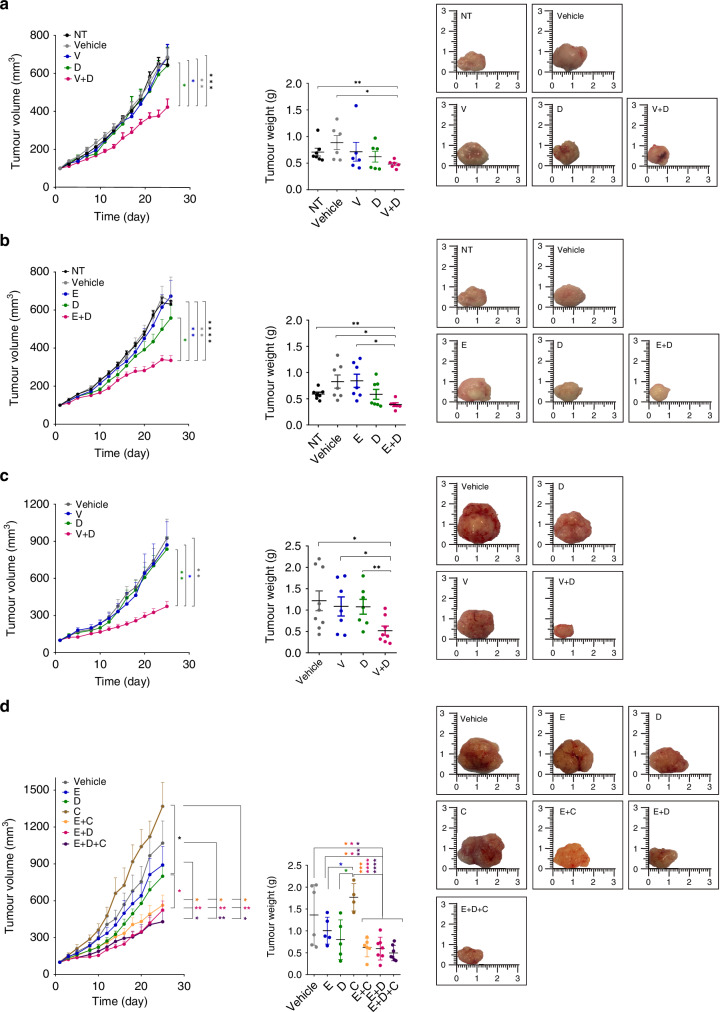
Inhibition of SRC and BRAFV600E reduces tumor growth in BRAFm CRC in vivo CDX models. **a** HT29 CDX models after treatment with single agent SRCi dasatinib, BRAF inhibitor vemurafenib or their combinations with SRC inhibitor (*n* = 6 mice per group, except NT (*n* = 7)). **b** HT29 CDX models after treatment with single agent SRCi dasatinib, BRAF inhibitor encorafenib or their combinations with SRC inhibitor (*n* = 7 mice per group; except, E + D (*n* = 6)). **c** BRAFm CRC PDX #1 treated with vehicle (VEH, control, *n* = 9), vemurafenib (*n* = 7), dasatinib (*n* = 7), vemurafenib and dasatinib (*n* = 8). **d** PDX #2 treated with vehicle (VEH, control, *n* = 6), encorafenib (*n* = 5), dasatinib (*n* = 5), cetuximab (*n* = 4), encorafenib and cetuximab (*n* = 6), encorafenib and dasatinib (*n* = 7), and the triplet combination of encorafenib, cetuximab and dasatinib (*n* = 7). Graphs represent: tumor volumes determined at indicated time points after treatments and last day tumor weight. Image of one representative tumor for each group after finishing the treatment were also shown. Data are represented as the mean ± SEM. Significance was considered for *P < 0.05, *P < 0.01 and **P < 0.001. NT non-treated, VEH vehicle, V vemurafenib, E encorafenib, D dasatinib, C cetuximab.

We further investigated the potential antitumor effect of the combination of dasatinib and BRAF inhibitors first in BRAF^V600E^ CRC HT29 CDX models (Fig. 6a, b) and then in two PDX mouse models (Fig. 6c, d). Previously (Supplementary Fig. 9), we confirmed both PDX maintained the same morphological, IHC and molecular pattern as the original patient tumor sample (Supplementary Fig. 9). Mice were treated with single agent dasatinib and BRAFi (vemurafenib or encorafenib), and with dual SRC and BRAF inhibition. As anticipated, BRAFi or SRCi alone were largely ineffective in controlling tumor growth. In contrast, significantly smaller tumors were observed when all CDX and PDX models were treated with the combination of both inhibitors as compared to those treated with monotherapies or vehicle (Fig. 6a–d).

Currently, the combination of encorafenib (BRAFi) and cetuximab (EGFRi) is the only EMA- and FDA-approved molecularly targeted regimen for patients with BRAF^V600E^ CRC [17, 18]. Consequently, we next considered whether the efficacy of BRAF and EGFR targeting could be improved by the addition of SRCi in vivo. To that aim, we tested combinations of BRAFi, EGFRi and/or SRCi in PDX #2. We observed that doublet combinations of encorafenib (E) plus dasatinib (D) and encorafenib (E) plus cetuximab (C), as well as the triplet combination E + D + C, resulted in significantly improved tumor growth inhibition compared to vehicle and monotherapies. However, no significant differences were found when the triplet therapy was compared to dual BRAF and EGFR inhibition or BRAF and SRC inhibition (Fig. 6d).

Consistent with our prior results, both combination treatments (D and V or E) in CDX mouse models led to a reduction of pSRC protein levels, which was accompanied by a decrease of pJUN levels. Moreover, the dual inhibition strategy downregulated pEGFR and p-ERK protein levels, demonstrating an effective suppression of SRC and MAPK pathways that could be responsible for the observed inhibition of tumor growth (Supplementary Fig. 10).

Of note, in vivo analysis of mice treated with dasatinib (D) alone further revealed an increase in pERK levels observed in some mice compared to vehicle-treated controls (Supplementary Fig. 11), highlighting the complexity of SRC signaling and the need for further investigation into the molecular mechanisms underlying this effect.

## Discussion

*BRAF*^V600E^ mutations are present in around 10% of advanced CRC patients and lead to constitutive activation of the MAPK pathway, drug resistance and a poor prognosis [1–4]. BRAF inhibitors have proven ineffective as single agents due to adaptive feedback re-activation of the MAPK and other signaling pathways [5–13, 38]. Combined EGFR and BRAF blockade proved synthetic lethality in preclinical models of BRAF^V600E^ CRC and modestly improves survival of patients with advanced disease [7, 8, 15–17]. Our work focused on unraveling other mediators of compensatory resistance to BRAF inhibition, and identified SRC as a pivotal signal transduction node involved in scape mechanisms to BRAF-targeted therapy in preclinical models of BRAF^V600E^ CRC. Our data demonstrates SRC modulates oncogenic capacities such as proliferation, colony formation and migration in BRAF^V600E^ CRC cell lines and CDX models. We also uncovered SRC mediates resistance to BRAFi and, more importantly, targeting SRC restores sensitivity to BRAFi in this context. Indeed, the combination of SRCi (dasatinib) and BRAFi (vemurafenib or encorafenib) results in a synergistic antitumor effect in BRAF^V600E^ CRC in vitro and in vivo models (CDX and PDX). This novel combination involves drugs that have been approved for the treatment of different solid tumors and are thus commercially available and safe to use in humans, paving the way for a rapid translation into clinical practice in a subgroup of CRC patients with a particularly dismal prognosis.

C-*SRC* was the first proto-oncogene discovered in the vertebrate genome [19–22]. Aberrant SRC activation has been associated with a more aggressive phenotype and a worse prognosis in CRC patients [27, 28, 39]. Indeed, prior work from our group demonstrated, in a large cohort of 487 patients with stage II-III colon cancer, that high pSRC expression in tumors is significantly associated with an increased risk of recurrence (5-year disease-free survival (DFS): 39% versus 63% for patients with high versus low pSRC expression; HR, 0.556; *P* = 0.005) and death (5-year OS: 58% versus 74% for patients with high versus low pSRC expression; HR, 0.55; *P* = 0.02) [27]. The present study confirms in BRAF^V600E^ CRC preclinical models that SRC is involved in the regulation of different oncogenic capacities. In fact, previous results of our group described the role of SRC in the resistance to platinum agents, and showed inhibition of SRC was able to restore sensitivity to oxaliplatin in orthotopic CRC PDX models [29]. In the present study, we observed that dasatinib efficiently inhibits SRC activation in a dose and time-dependent manner and decreases cell proliferation and migration in in vitro assays. Despite this antitumor effect observed in vitro, the evaluation of dasatinib as a single agent in small cohorts of molecularly unselected, refractory metastatic CRC patients failed to show clinically meaningful results in clinical trials [40]. We recognize that while dasatinib primarily targets SFKs and BCR-ABL, it also inhibits other kinases including c-KIT, PDGFRβ, EPHA2, among others [41]. Thus, non-specific effects beyond SRC inhibition cannot be ruled out.

Moreover, previous studies have reported that ATP-competitive inhibitors, such as dasatinib, can paradoxically activate SRC and its downstream signaling pathways [42]. This phenomenon occurs when inhibitor binding induces conformational changes in SRC, facilitating the formation of an active SRC-FAK complex that, upon inhibitor dissociation, phosphorylates FAK and initiates ERK signaling. Additionally, acquired drug-induced mutations in SRC that reduce inhibitor affinity may enhance FAK and ERK phosphorylation. Therefore, the observed increase in pERK in some mice treated with dasatinib monotherapy in our in vivo experiment (Supplementary Fig. S11) may be the consequence of this paradoxical SRC activation. In any case, dasatinib antitumor effect in combination with BRAFi remains evident and of relevant potential translational value that deserves to be clinically assessed in BRAF^V600E^ CRC patients.

But the most relevant finding of our study is that upregulation of SRC induced by BRAFi unravels a potentially druggable adaptive mechanism of resistance to BRAFi. Rapid EGFR feedback activation has been established as one of the mechanisms of intrinsic resistance to BRAFi in BRAF^V600E^ CRC, which does not occur in BRAF^V600E^ melanoma and explains the sensitivity of the latest to BRAF inhibitors in the clinic [7, 8]. SRC plays a role in EGFR transactivation, acting as an upstream effector of EGFR in the feedback loop [20, 43]. In addition to MAPK, SRC kinases can directly modulate many downstream targets including and c-MYC [19, 21, 22, 44], related to drug resistance and tumor cell survival. Thus, inhibition of SRC could have a broad impact on tumor progression.

Consistent with the upregulation of pSRC expression after BRAF inhibition in BRAF^V600E^ CRC cells, we found that SRC depletion resulted in a significantly better response to the BRAF inhibitors vemurafenib and encorafenib, pointing to SRC as one of the main critical players in the resistance mechanisms to BRAF inhibition in BRAF^V600E^ CRC. Moreover, dual pharmacological inhibition of SRC and BRAF caused a marked decrease of oncogenic capacities such as proliferation, migration and clonogenicity in a variety of cell lines with several inhibitors of the same class, and also resulted in a robust induction of apoptosis and cell cycle arrest as compared to treatment with these drugs as single agents.

Furthermore, we demonstrated in both BRAF^V600E^ CRC cell line xenograft mouse models and PDXs that dual inhibition of SRC and BRAF synergistically reduced tumor growth compared with single-drug therapy. More importantly, the proposed combined therapeutic strategy showed the same efficacy in CRC PDX as the recent FDA and EMA approved therapy for BRAF^V600E^ CRC of encorafenib (BRAFi) and cetuximab (anti-EGFR) [18]. These results further highlight the efficacy of our proposed novel combination with SRCi and BRAFi, which represents an alternative therapeutic strategy for this subgroup of patients that have limited treatment options, of particular interest for patients resistant or intolerant to EGFR-targeted therapy.

Moreover, the efficacy of the combination therapy is independent of the microsatellite instability (MSI) status, since the results found in the in vivo models, CDX (MSS) and PDX (MSI), showed the same synergistic effect, highlighting its potential utility as a treatment option irrespective of MSI context.

Consistent with our data, the recently published work of Ruiz-Saenz et al. [45] also showed that SRC plays a major role in adaptive resistance to BRAF inhibition in BRAF^V600E^ CRC. Their study revealed that targeting SRC and BRAF had an equivalent or better effect on tumor growth inhibition compared to targeting EGFR and BRAF, similar to our observations. However, they did not evaluate the therapy with cetuximab, the EGFR inhibitor more commonly used in clinical setting and recently approved, which more faithfully recapitulates the patient context.

Our data are also consistent with emerging studies in other solid tumors harboring the *BRAF*^V600E^ mutation, such as thyroid cancer or melanoma [34, 46, 47]. Our hypothesis is also further reinforced by published reports in BRAF^V600E^ melanoma that showed activation of the SRC pathway by Aryl hydrocarbon Receptor (AhR) promotes the acquisition of an invasive and aggressive phenotype resistant to front-line BRAF inhibitors [34]. Additionally, studies in breast cancer revealed SRC as a common node downstream of multiple resistance pathways [44]. Altogether, these data highlight the important role of SRC as a master regulator of multiple growth stimulatory and stress pathways that are involved, among other processes, in resistance to different drugs, including BRAF inhibitors, further supporting SRC as a promising targetable vulnerability that deserves to be explored in the clinic.

To explore the molecular mechanisms involved in the synergistic effect of dual SRC and BRAF inhibition, we performed a proteome profile of human phospho-kinases in BRAF^V600E^ cells treated with dasatinib and/or vemurafenib, and observed an upregulation of c-JUN phosphorylation upon exposure to combined SRCi and BRAFi as compared to single agents. C-JUN is a proto-oncogene which is regulated by c-JUN N-terminal Kinases (JNKs) [48, 49], a sub-family of MAPKs activated by environmental stress, inflammatory cytokines and growth factors. Different studies in several human neoplasms [50–53], including CRC [54], showed higher c-JUN expression and activation in tumors compared to normal tissue. However, the specific role of c-JUN in CRC has not been fully elucidated. Our data showed that inhibition of the JNK/c-Jun pathway in BRAF^V600E^ CRC cell lines resulted in reduced cell proliferation and increased apoptosis, whereas its overexpression showed the opposite effect, unveiling a role of c- JUN in cancer cell survival. We also discovered that c-JUN inhibition combined with SRCi and BRAFi significantly potentiates cell apoptosis in BRAF^V600E^ cells, demonstrating the important role c-JUN plays in BRAF^V600E^ CRC. We hypothesized that c-JUN upregulation upon dual BRAF and SRC inhibition in BRAF^V600E^ CRC cells may be an adaptive cellular response inducing resistance. This is consistent with BRAF^V600E^ melanoma studies that demonstrated a role for the JNK/c-Jun signaling pathway in vemurafenib adaptation and resistance to drug-induced apoptosis [55]. Additionally, c-JUN induces an EMT-like phenotype switch that may be responsible for the adaptive resistance to BRAF-MEK-ERK pathway inhibition in BRAF^V600E^ melanoma cells [56].

Overall, our study identifies, for the first time, c-JUN as a targetable mechanism of adaptive resistance to BRAF inhibition in BRAF^V600E^ CRC paving the way to the development of future combinational therapeutic strategies incorporating JNKi. These strategies may eventually expand treatment options and improve patient’s clinical outcomes.

In conclusion, our results demonstrate that SRC regulates oncogenic capacities and mediates resistance to targeted therapy in BRAF^V600E^ CRC. We also show that targeting SRC overcomes resistance to BRAF inhibition. The novel combination we developed, consisting of SRCi (dasatinib) and BRAFi (vemurafenib and encorafenib), results in synergistic anticancer activity in BRAF^V600E^ CRC, both in vitro and in vivo. Although the mechanisms involved in this synergism remain to be elucidated, our results suggest that cross-talk between MAPK and JNK signaling plays a relevant role. An improved understanding on how these signaling pathways are rewired offers potential new targets to optimize therapy. These promising results warrant to be further assessed in the clinic as they pave the way to expand treatment options for BRAF^V600E^ mCRC patients and eventually improve survival of this aggressive subpopulation of CRC.

## Supplementary information

Supplemetary data

## Data Availability

Data generated in this study are available within the article and its supplementary data files and are available upon request from the corresponding author.

## References

[1] Morgan E, Arnold M, Gini A, Lorenzoni V, Cabasag CJ, Laversanne M, et al. Global burden of colorectal cancer in 2020 and 2040: incidence and mortality estimates from GLOBOCAN. Gut. 2023;72:338–44. 36604116 10.1136/gutjnl-2022-327736

[2] Davies H, Bignell GR, Cox C, Stephens P, Edkins S, Clegg S, et al. Mutations of the BRAF gene in human cancer. Nature. 2002;417:949–54. 12068308 10.1038/nature00766

[3] Morris V, Overman MJ, Jiang ZQ, Garrett C, Agarwal S, Eng C, et al. Progression-free survival remains poor over sequential lines of systemic therapy in patients with BRAF-mutated colorectal cancer. Clin Colorectal Cancer. 2014;13:164–71. 25069797 10.1016/j.clcc.2014.06.001 PMC4266576

[4] Poulikakos PI, Sullivan RJ, Yaeger R. Molecular pathways and mechanisms of BRAF in cancer therapy. Clin Cancer Res. 2022;28:4618–28. 35486097 10.1158/1078-0432.CCR-21-2138 PMC9616966

[5] Kopetz S, Desai J, Chan E, Hecht JR, O’Dwyer PJ, Maru D, et al. Phase II pilot study of vemurafenib in patients with metastatic *BRAF* -mutated colorectal cancer. JCO. 2015;33:4032–8. 10.1200/JCO.2015.63.2497 PMC4669589 26460303

[6] Hyman DM, Puzanov I, Subbiah V, Faris JE, Chau I, Blay JY, et al. Vemurafenib in multiple nonmelanoma cancers with BRAF V600 mutations. N Engl J Med. 2015;373:726–36. 26287849 10.1056/NEJMoa1502309 PMC4971773

[7] Corcoran RB, Ebi H, Turke AB, Coffee EM, Nishino M, Cogdill AP, et al. EGFR-mediated reactivation of MAPK signaling contributes to insensitivity of *BRAF* -mutant colorectal cancers to RAF inhibition with vemurafenib. Cancer Discov. 2012;2:227–35. 22448344 10.1158/2159-8290.CD-11-0341 PMC3308191

[8] Prahallad A, Sun C, Huang S, Di Nicolantonio F, Salazar R, Zecchin D, et al. Unresponsiveness of colon cancer to BRAF(V600E) inhibition through feedback activation of EGFR. Nature. 2012;483:100–3. 22281684 10.1038/nature10868

[9] Yang H, Higgins B, Kolinsky K, Packman K, Bradley WD, Lee RJ, et al. Antitumor activity of BRAF inhibitor vemurafenib in preclinical models of BRAF-mutant colorectal cancer. Cancer Res. 2012;72:779–89. 22180495 10.1158/0008-5472.CAN-11-2941

[10] Mao M, Tian F, Mariadason JM, Tsao CC, Lemos R, Dayyani F, et al. Resistance to BRAF inhibition in BRAF-mutant colon cancer can be overcome with PI3K inhibition or demethylating agents. Clin Cancer Res. 2013;19:657–67. 23251002 10.1158/1078-0432.CCR-11-1446 PMC3563727

[11] Hanrahan AJ, Chen Z, Rosen N, Solit DB. BRAF - a tumour-agnostic drug target with lineage-specific dependencies. Nat Rev Clin Oncol. 2024;21:224–47. 38278874 10.1038/s41571-023-00852-0 PMC11857949

[12] Poulikakos PI, Persaud Y, Janakiraman M, Kong X, Ng C, Moriceau G, et al. RAF inhibitor resistance is mediated by dimerization of aberrantly spliced BRAF(V600E). Nature. 2011;480:387–90. 22113612 10.1038/nature10662 PMC3266695

[13] Johannessen CM, Boehm JS, Kim SY, Thomas SR, Wardwell L, Johnson LA, et al. COT drives resistance to RAF inhibition through MAP kinase pathway reactivation. Nature. 2010;468:968–72. 21107320 10.1038/nature09627 PMC3058384

[14] Corcoran RB, Atreya CE, Falchook GS, Kwak EL, Ryan DP, Bendell JC, et al. Combined BRAF and MEK inhibition with dabrafenib and trametinib in *BRAF* V600–mutant colorectal cancer. JCO. 2015;33:4023–31. 10.1200/JCO.2015.63.2471 PMC4669588 26392102

[15] Corcoran RB, André T, Atreya CE, Schellens JHM, Yoshino T, Bendell JC, et al. Combined BRAF, EGFR, and MEK inhibition in patients with BRAFV600E-mutant colorectal cancer. Cancer Discov. 2018;8:428–43. 29431699 10.1158/2159-8290.CD-17-1226 PMC5882509

[16] Van Geel RMJM, Tabernero J, Elez E, Bendell JC, Spreafico A, Schuler M, et al. A phase Ib dose-escalation study of encorafenib and cetuximab with or without alpelisib in metastatic BRAF-mutant colorectal cancer. Cancer Discov. 2017;7:610–9. 28363909 10.1158/2159-8290.CD-16-0795 PMC5546207

[17] Kopetz S, Grothey A, Yaeger R, Van Cutsem E, Desai J, Yoshino T, et al. Encorafenib, binimetinib, and cetuximab in *BRAF* V600E–mutated colorectal cancer. N Engl J Med. 2019;381:1632–43. 31566309 10.1056/NEJMoa1908075

[18] Tabernero J, Grothey A, Van Cutsem E, Yaeger R, Wasan H, Yoshino T, et al. Encorafenib plus cetuximab as a new standard of care for previously treated *BRAF* V600E–mutant metastatic colorectal cancer: updated survival results and subgroup analyses from the BEACON study. JCO. 2021;39:273–84. 10.1200/JCO.20.02088 PMC8078423 33503393

[19] Martellucci S, Clementi L, Sabetta S, Mattei V, Botta L, Angelucci A. Src family kinases as therapeutic targets in advanced solid tumors: what we have learned so far. Cancers. 2020;12:1448. 32498343 10.3390/cancers12061448 PMC7352436

[20] Gargalionis AN, Karamouzis MV, Papavassiliou AG. The molecular rationale of Src inhibition in colorectal carcinomas. Int J Cancer. 2014;134:2019–29. 23733480 10.1002/ijc.28299

[21] Wheeler DL, Iida M, Dunn EF. The role of Src in solid tumors. Oncologist. 2009;14:667–78. 19581523 10.1634/theoncologist.2009-0009 PMC3303596

[22] Summy JM, Gallick GE. Src family kinases in tumor progression and metastasis. Cancer Metastasis Rev. 2003;22:337–58. 12884910 10.1023/a:1023772912750

[23] Cartwright CA, Meisler AI, Eckhart W. Activation of the pp60c-src protein kinase is an early event in colonic carcinogenesis. Proc Natl Acad Sci USA. 1990;87:558–62. 2105487 10.1073/pnas.87.2.558 PMC53304

[24] Han NM, Curley SA, Gallick GE. Differential activation of pp60(c-src) and pp62(c-yes) in human colorectal carcinoma liver metastases. Clin Cancer Res. 1996;2:1397–404. 9816313

[25] Talamonti MS, Roh MS, Curley SA, Gallick GE. Increase in activity and level of pp60c-src in progressive stages of human colorectal cancer. J Clin Invest. 1993 ;91:53–60. 7678609 10.1172/JCI116200 PMC329994

[26] Termuhlen PM, Curley SA, Talamonti MS, Saboorian MH, Gallick GE. Site-specific differences in pp60c-src activity in human colorectal metastases. J Surg Res. 1993;54:293–8. 7687314 10.1006/jsre.1993.1046

[27] Martínez-Pérez J, Lopez-Calderero I, Saez C, Benavent M, Limon ML, Gonzalez-Exposito R, et al. Prognostic relevance of Src activation in stage II-III colon cancer. Hum Pathol. 2017;67:119–25. 28601656 10.1016/j.humpath.2017.05.025

[28] Allgayer H, Boyd DD, Heiss MM, Abdalla EK, Curley SA, Gallick GE. Activation of Src kinase in primary colorectal carcinoma: An indicator of poor clinical prognosis. Cancer. 2002;94:344–51. 11900220 10.1002/cncr.10221

[29] Perez M, Lucena-Cacace A, Marín-Gómez LM, Padillo-Ruiz J, Robles-Frias MJ, Saez C, et al. Dasatinib, a Src inhibitor, sensitizes liver metastatic colorectal carcinoma to oxaliplatin in tumors with high levels of phospho-Src. Oncotarget. 2016;7:33111–24. 27105527 10.18632/oncotarget.8880 PMC5078079

[30] Simpkins F, Jang K, Yoon H, Hew KE, Kim M, Azzam DJ, et al. Dual Src and MEK inhibition decreases ovarian cancer growth and targets tumor initiating stem-like cells. Clin Cancer Res. 2018;24:4874–86. 29959144 10.1158/1078-0432.CCR-17-3697 PMC6557165

[31] Chen Y, Alvarez EA, Azzam D, Wander SA, Guggisberg N, Jordà M, et al. Combined Src and ER blockade impairs human breast cancer proliferation in vitro and in vivo. Breast Cancer Res Treat. 2011;128:69–78. 20669046 10.1007/s10549-010-1024-7

[32] Dunn EF, Iida M, Myers RA, Campbell DA, Hintz KA, Armstrong EA, et al. Dasatinib sensitizes KRAS mutant colorectal tumors to cetuximab. Oncogene. 2011;30:561–74. 20956938 10.1038/onc.2010.430 PMC3025039

[33] Girotti MR, Lopes F, Preece N, Niculescu-Duvaz D, Zambon A, Davies L, et al. Paradox-breaking RAF inhibitors that also target SRC are effective in drug-resistant BRAF mutant melanoma. Cancer cell Cancer cell 2015;27:85–96. 25500121 10.1016/j.ccell.2014.11.006 PMC4297292

[34] Paris A, Tardif N, Baietti FM, Berra C, Leclair HM, Leucci E, et al. The AhR‐SRC axis as a therapeutic vulnerability in BRAFi‐resistant melanoma. EMBO Mol Med. 2022;14:e15677. 36305167 10.15252/emmm.202215677 PMC9728058

[35] Chou TC. Drug combination studies and their synergy quantification using the Chou-Talalay method. Cancer Res. 2010;70:440–6. 20068163 10.1158/0008-5472.CAN-09-1947

[36] Liang W, Liu D, Wu J. c-JUN-induced upregulation of LINC00174 contributes to colorectal cancer proliferation and invasion through accelerating USP21 expression. Cell Biol Int. 2023;47:1782–98. 37434557 10.1002/cbin.12069

[37] Moyano AJ, Racca AC, Soria G, Saka HA, Andreoli V, Smania AM, et al. c-Jun proto-oncoprotein plays a protective role in lung epithelial cells exposed to staphylococcal α-toxin. Front Cell Infect Microbiol. 2018;8:170. 29888211 10.3389/fcimb.2018.00170 PMC5981160

[38] Guerrero P, Albarrán V, San Román M, González-Merino C, García De Quevedo C, Moreno J, et al. BRAF inhibitors in metastatic colorectal cancer and mechanisms of resistance: a review of the literature. Cancers. 2023;15:5243. 37958416 10.3390/cancers15215243 PMC10649848

[39] Yeatman TJ. A renaissance for SRC. Nat Rev Cancer. 2004;4:470–80. 15170449 10.1038/nrc1366

[40] Sharma MR, Wroblewski K, Polite BN, Knost JA, Wallace JA, Modi S, et al. Dasatinib in previously treated metastatic colorectal cancer: a phase II trial of the University of Chicago Phase II Consortium. Invest N. Drugs. 2012;30:1211–5. 10.1007/s10637-011-9681-x PMC4317401 21552992

[41] Hantschel O, Rix U, Superti-Furga G. Target spectrum of the BCR-ABL inhibitors imatinib, nilotinib and dasatinib. Leuk Lymphoma. 2008;49:615–9. 18398720 10.1080/10428190801896103

[42] Higuchi M, Ishiyama K, Maruoka M, Kanamori R, Takaori-Kondo A, Watanabe N. Paradoxical activation of c-Src as a drug-resistant mechanism. Cell Rep. 2021;34:108876. 33761359 10.1016/j.celrep.2021.108876

[43] Kopetz S. Targeting Src and epidermal growth factor receptor in colorectal cancer: rationale and progress into the clinic. Gastrointest cancer res: Gcr 2007;1:S37–41. 19360146 PMC2666842

[44] Zhang S, Huang WC, Li P, Guo H, Poh SB, Brady SW, et al. Combating trastuzumab resistance by targeting SRC, a common node downstream of multiple resistance pathways. Nat Med. 2011;17:461–9. 21399647 10.1038/nm.2309 PMC3877934

[45] Ruiz-Saenz A, Atreya CE, Wang C, Pan B, Dreyer CA, Brunen D, et al. A reversible SRC-relayed COX2 inflammatory program drives resistance to BRAF and EGFR inhibition in BRAFV600E colorectal tumors. Nat Cancer. 2023;4:240–56. 36759733 10.1038/s43018-022-00508-5 PMC9970872

[46] Borre PV, Gunda V, McFadden DG, Sadow PM, Varmeh S, Bernasconi M, et al. Combined BRAFV600E- and SRC-inhibition induces apoptosis, evokes an immune response and reduces tumor growth in an immunocompetent orthotopic mouse model of anaplastic thyroid cancer. Oncotarget. 2014;5:3996–4010. 24994118 10.18632/oncotarget.2130 PMC4147301

[47] Ghosh C, Kumar S, Kushchayeva Y, Gaskins K, Boufraqech M, Wei D, et al. A combinatorial strategy for targeting *BRAF* V600E-mutant cancers with BRAFV600E inhibitor (PLX4720) and tyrosine kinase inhibitor (ponatinib). Clin Cancer Res. 2020;26:2022–36. 31937621 10.1158/1078-0432.CCR-19-1606 PMC8606228

[48] Zhou YY, Li Y, Jiang WQ, Zhou LF. MAPK/JNK signalling: a potential autophagy regulation pathway. Biosci Rep. 2015;35:e00199. 26182361 10.1042/BSR20140141 PMC4613668

[49] Bohmann D, Bos TJ, Admon A, Nishimura T, Vogt PK, Tjian R. Human proto-oncogene c-jun encodes a DNA binding protein with structural and functional properties of transcription factor AP-1. Science. 1987;238:1386–92. 2825349 10.1126/science.2825349

[50] Mathas S, Hinz M, Anagnostopoulos I, Krappmann D, Lietz A, Jundt F, et al. Aberrantly expressed c-Jun and JunB are a hallmark of Hodgkin lymphoma cells, stimulate proliferation and synergize with NF-kappa. B EMBO J. 2002;21:4104–13. 12145210 10.1093/emboj/cdf389 PMC126136

[51] Rangatia J, Vangala RK, Singh SM, Peer Zada AA, Elsässer A, Kohlmann A, et al. Elevated c-Jun expression in acute myeloid leukemias inhibits C/EBPalpha DNA binding via leucine zipper domain interaction. Oncogene. 2003;22:4760–4. 12879022 10.1038/sj.onc.1206664

[52] Vleugel MM, Greijer AE, Bos R, Van Der Wall E, Van Diest PJ. c-Jun activation is associated with proliferation and angiogenesis in invasive breast cancer. Hum Pathol. 2006;37:668–74. 16733206 10.1016/j.humpath.2006.01.022

[53] Szabo E, Riffe ME, Steinberg SM, Birrer MJ, Linnoila RI. Altered cJUN expression: an early event in human lung carcinogenesis. Cancer Res. 1996;56:305–15. 8542585

[54] Wang H, Birkenbach M, Hart J. Expression of Jun family members in human colorectal adenocarcinoma. Carcinogenesis 2000;21:1313–7. 10874008

[55] Fallahi-Sichani M, Moerke NJ, Niepel M, Zhang T, Gray NS, Sorger PK. Systematic analysis of BRAF(V600E) melanomas reveals a role for JNK/c-Jun pathway in adaptive resistance to drug-induced apoptosis. Mol Syst Biol. 2015;11:797. 25814555 10.15252/msb.20145877 PMC4380931

[56] Ramsdale R, Jorissen RN, Li FZ, Al-Obaidi S, Ward T, Sheppard KE, et al. The transcription cofactor c-JUN mediates phenotype switching and BRAF inhibitor resistance in melanoma. Sci Signal. 2015;8:ra82. 10.1126/scisignal.aab1111 26286024

