# Supplementary material for: Co-targeting SRC overcomes resistance to BRAF inhibitors in colorectal cancer
Source: Br J Cancer. 2025 Jun 6;133(3):404–19. doi: 10.1038/s41416-025-03058-6 (PMC12322152; doi:10.1038/s41416-025-03058-6)
Supplement: Supplementary file 1 — Supplemetary data [file 41416_2025_3058_MOESM1_ESM.pdf]

Figure S1.

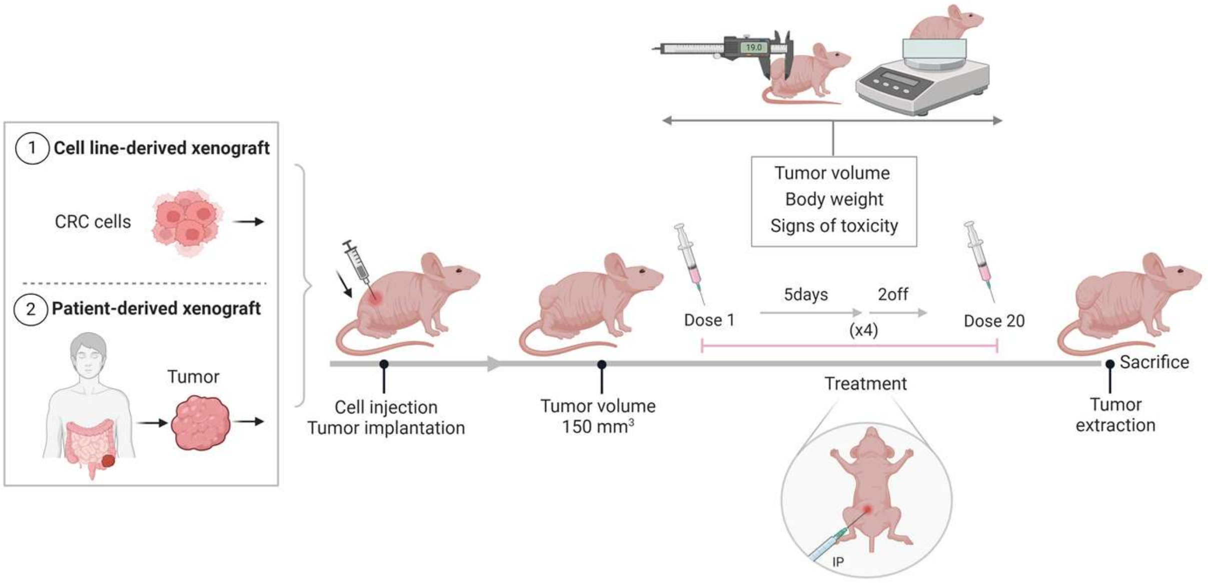

Scheme summarizing the development of CDX and PDX mouse experiments. Human tumor cell lines (1) or tumor pieces (2) are subcutaneously injected into the flank of athymic nude mice. When the tumor reaches a specific volume (150 mm^3), treatment of mice starts (consisting of 20 doses, except Cetuximab, 8). During this period, mice are observed and weighted to monitor potential toxicity and tumors are regularly measured. Once the treatment regimen is completed, mice are sacrificed, and tumor is extracted for further analysis. IP, intraperitoneally.

Figure S2.

A

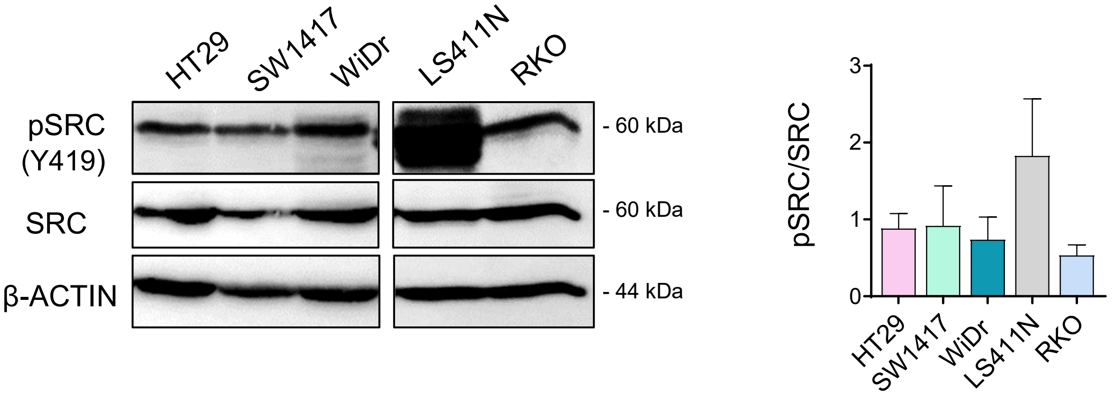

B

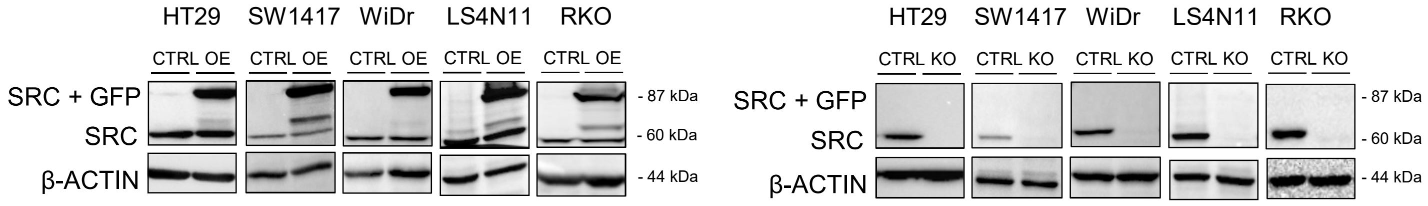

SRC expression in a panel of 5 CRC BRAFm cell lines. A. Representative WB of p-SRC and total SRC expression (left). β-actin used as a loading control. Molecular weight/size markers are indicated in kilodaltons (kDa), and the band densitometry representation of these data is shown on the right. N = 3 biologically independent experiments. **B.** WB analysis showing *in vitro* validation of SRC overexpression (left) and SRC depletion (right) in BRAFm CRC cell lines. Green fluorescent protein (GFP) is used as a reporter in the plasmid for the transfection. SRC + GFP label indicates the presence of a SRC-GFP fusion. β-actin levels were used as a loading control. Molecular weight/size markers are indicated on the right (kDa). Images are representative of *n* = 3 replicates. CTRL, control. OE, overexpression. KO, knock-out.

Figure S3.

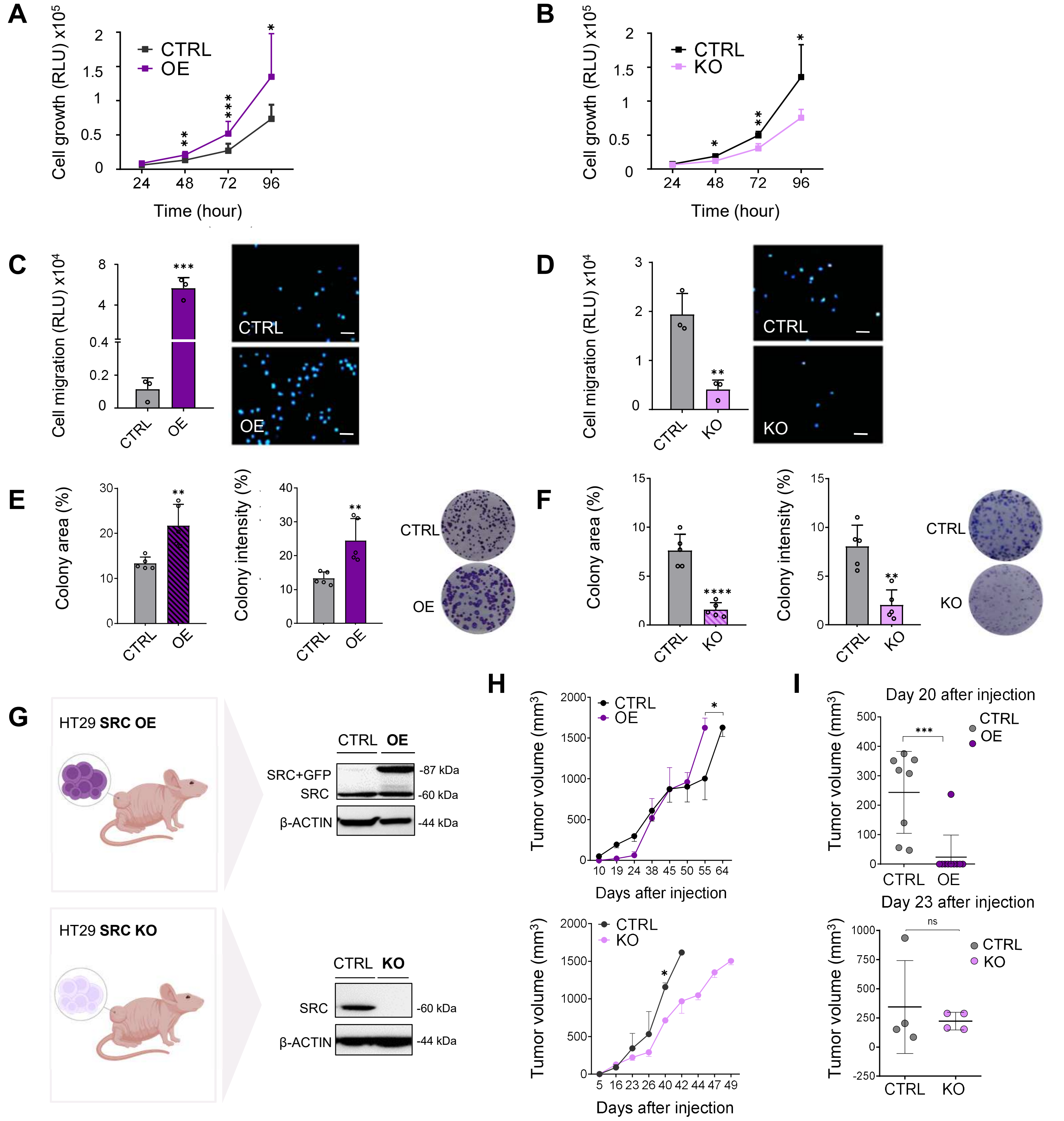

SRC regulates proliferation, migration and clonogenicity abilities in HT29 BRAFm CRC. **A, B.** CellTiter-Glo assay was used to measure cell viability at the different indicated time points in SRC OE (represented in purple color) and SRC KO cells (in lilac color). **C, D.** Transwell assay for cell migration in SRC OE and SRC KO cells. Transmigrated cells were quantified by cell titter (left graph), and representative images of the transwell after DAPI staining (right) are shown. **E, F.** CFA for the clonogenic capacity of SRC OE (**E**) and SRC KO cells (**F**). Statistical results of colony area and colony intensity, measured by ImageJ software. Representative images of the colony formation assay after crystal violet staining (right). Data are the mean ± SD of 5 independent experiments. **G.** Schemes of tumor generation. **G.** Scheme of tumor generation. HT29 SRC OE (in purple color) and SRC KO cells (in lilac) and their respective controls were subcutaneously implanted in nude mice . Expression levels of SRC protein in tumors were evaluated by WB. β-actin levels were used as a loading control. Molecular weight/size markers are indicated on the right (kDa). **H.** Tumor volumes for HT29 SRC OE (upper part) and SRC KO cells (lower part) and control cell lines xenograft mouse models at indicated time points. Tumor volume was monitored three times a week and presented as a mean ± SEM. n = 8 mice for SRC OE and control tumors and n = 4 for SRC KO and CTRL mice. **I.** Tumor volumes 20 and 23 days after injection of HT29 SRC OE (purple, upper part) and SRC KO cells (lilac, lower part), respectively. Significance was considered for * P < 0.05, ** P < 0.01, *** P < 0.001. Ns, non-significant. CTRL, control. OE, overexpression. KO, knock-out.

Figure S4.

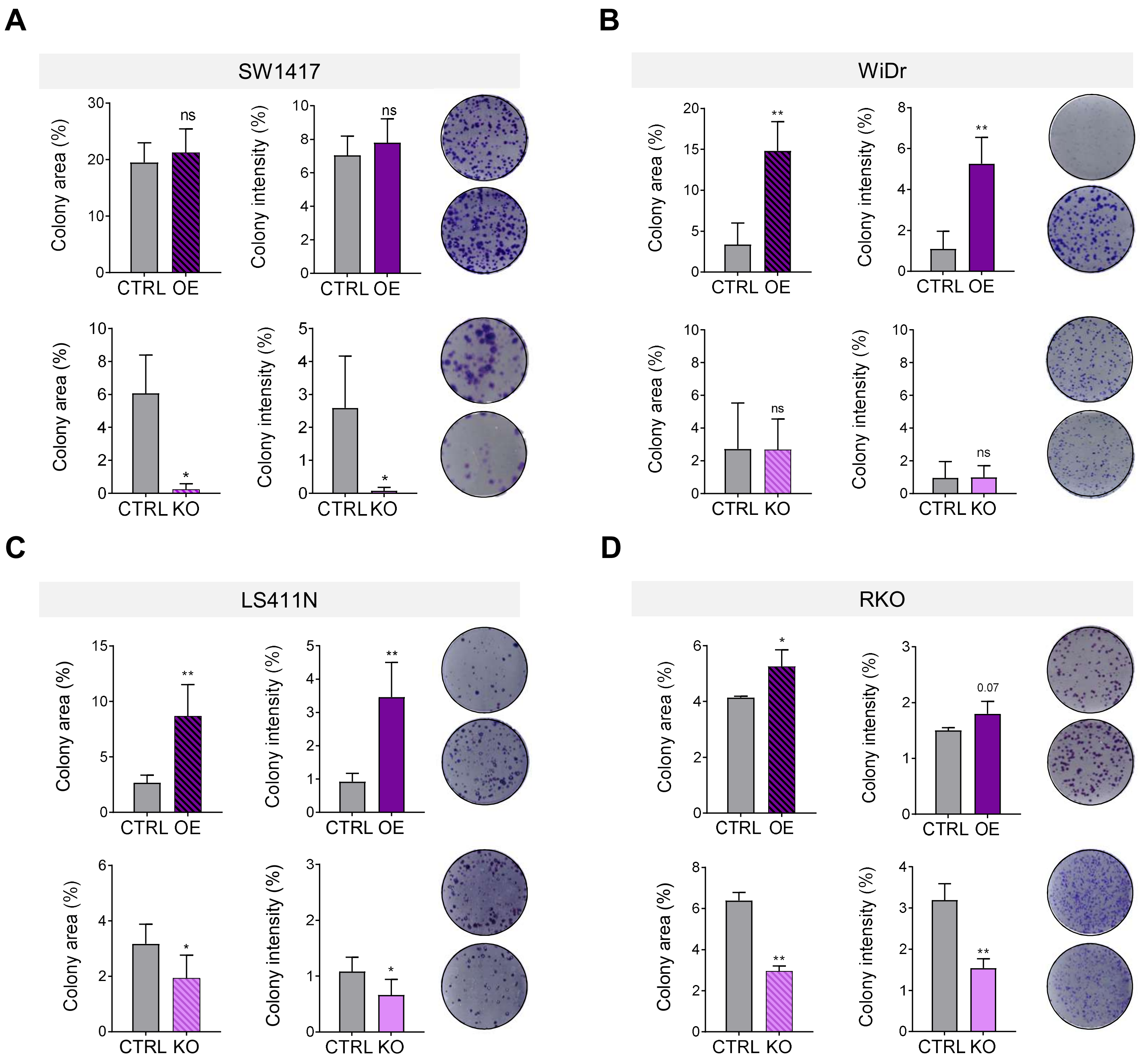

SRC expression modulates the colony formation capability in BRAFm cell lines. Left graphs represent the quantification of the area and intensity of the colonies formed by SRC OE (upper part, purple color) and SRC KO cell lines (lower part, lilac color) and their respective controls (gray color). On the right, representative images of the colonies in SW1417 (**A**), WiDr (**B**), LS411N (**C**) and RKO (**D**) BRAFm cell lines (CTRL colonies in the upper circle, OE/KO colonies in the lower circle). Data represent the mean ± SD of 3 independent experiments. Significance was considered for *$P < 0.05$* and **$P < 0.01$*. Ns, non-significant. CTRL, control. OE, overexpression. KO, knock-out.

Figure S5.

Depletion of SRC reverts resistance to BRAFi in the whole set of BRAFm CRC cell lines. A. Dose-response assay of CRC BRAFm cell lines treated with BRAFi (vemurafenib (left) and encorafenib (right)) for 72 h indicating the IC50 for each drug and cell line (mean ± SD of 3 biologically independent experiments). **B.** IC50 values of vemurafenib and encorafenib (72h) for each BRAFm CRC cell lines (OE and KO versus CTRL) (data represent the mean ± SD of 3 biologically independent experiments). **C.** Left, representative WBs for validation of SRC OE and SRC KO DIFI cell lines. β-actin used as a loading control. Molecular weight/size markers are indicated on the right (kDa). On the right, dose-response assay of SRC OE and KO DIFI cell line treated with vemurafenib or encorafenib for 72h (graphs are representative of 3 biologically independent experiments). GFP, green fluorescent protein. IC50, half-maximal inhibitory concentration. CTRL, control. OE, overexpression. KO, knock-out.

A

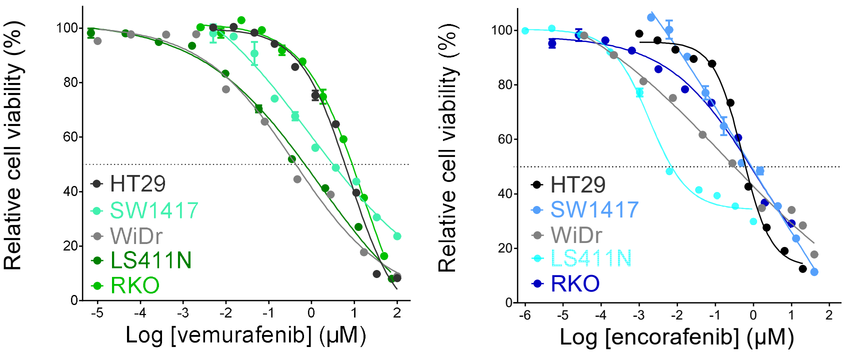

Cell line	IC50 VEMURAFENIB (μM)	IC50 ENCORAFENIB (μM)
HT29	7.500 ± 2.500	0.500 ± 0.300
SW1417	3.140 ± 1.970	0.120 ± 0.030
WiDr	0.530 ± 0.150	0.040 ± 0.030
LS411N	0.600 ± 0.200	0.001 ± 0.001
RKO	8.000 ± 1.700	0.700 ± 0.030

B

Cell line	IC50 Vemurafenib (μM)				IC50 Encorafenib (μM)			
	CTRL	OE	CTRL	KO	CTRL	OE	CTRL	KO
HT29	4.200 ± 2.800	25.000 ± 10.300	4.040 ± 2.300	1.200 ± 0.9000	0.350 ± 0.200	1.500 ± 0.400	7.000 ± 5.000	0.700 ± 0.330
SW1417	15.000 ± 2.500	20.000 ± 4.000	9.000 ± 4.000	8.800 ± 3.000	2.000 ± 0.800	9.000 ± 1.600	0.800 ± 0.200	0.700 ± 0.300
WiDr	0.030 ± 0.010	2.500 ± 0.700	0.080 ± 0.010	0.100 ± 0.010	0.002 ± 0.001	1.500 ± 0.500	0.010 ± 0.012	0.010 ± 0.001
LS411N	5.100 ± 0.570	5.750 ± 0.350	3.800 ± 1.700	1.600 ± 1.700	0.004 ± 0.003	0.005 ± 0.004	0.010 ± 0.010	0.021 ± 0.030
RKO	9.000 ± 5.000	13.000 ± 2.500	11.000 ± 4.500	6.000 ± 4.000	0.200 ± 0.200	0.800 ± 0.500	1.100 ± 0.700	0.500 ± 0.400

C

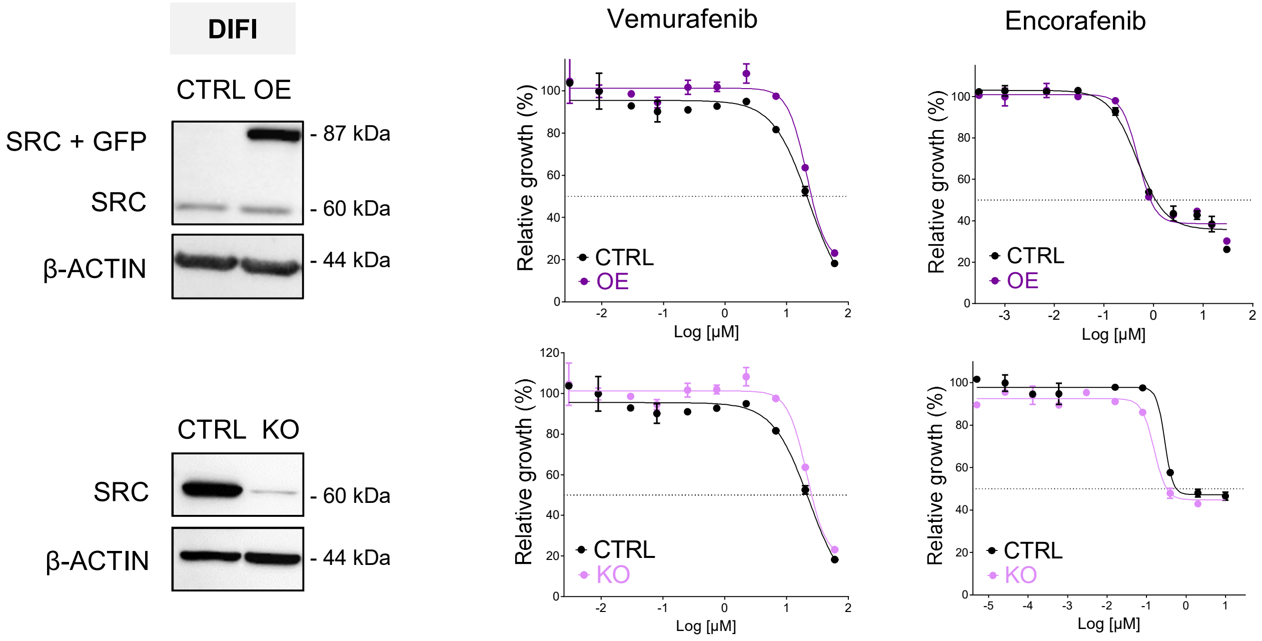

Figure S6.

A

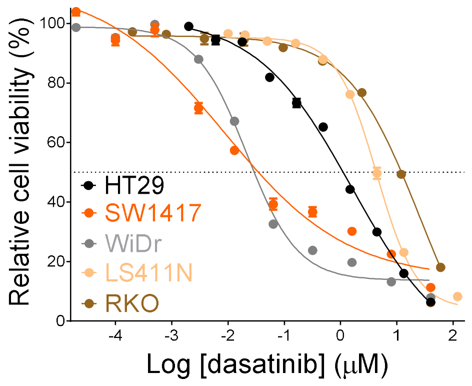

CELL LINE	IC50 DASATINIB (μM)
HT29	1.20 ± 0.25
SW1417	0.06 ± 0.05
WiDr	0.02 ± 0.01
LS411N	5.31 ± 1.30
RKO	11.00 ± 0.03

B

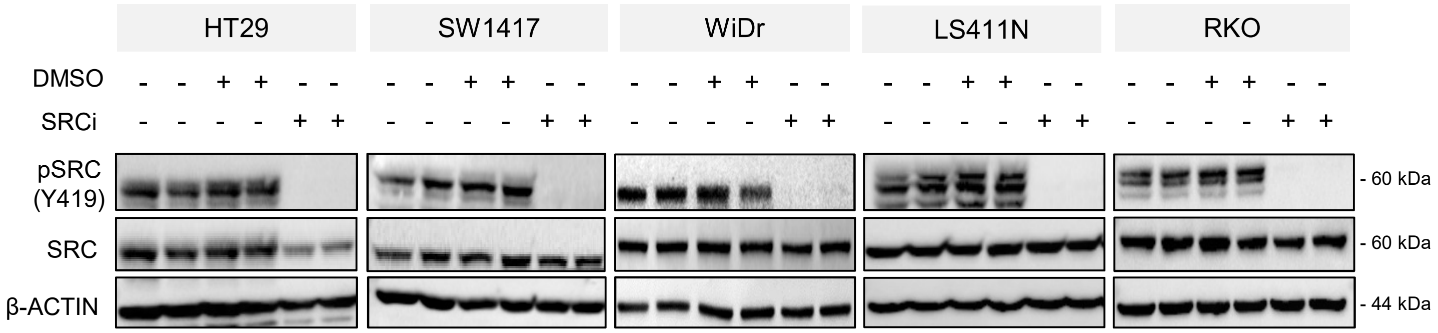

Dasatinib efficiently inhibits p-SRC in BRAFm cell lines. A. Dose-response curves of the indicated cell lines treated with increasing concentrations of dasatinib for 72h. Error bars represent the mean ± SD of 3 biologically independent experiments. On the right, IC50 values for each CRC cell line (mean ± SD of 3 biologically independent experiments**)**. **B.** Representative WB for CRC cell lines after treatment with dasatinib (IC50 values), DMSO (as vehicle) or no therapy for 24h. β-actin was used as a loading control. Molecular weight/size markers are indicated on the right (kDa). Images are representative of 3 independent experiments. IC50, half-maximal inhibitory concentration.

Figure S7.

CELL LINE	V + D (µM)	CI	E + D (µM)	CI
SW1417	0.080 µM + 0.001 µM	0.16	0.030 µM + 0.001 µM	0.26
SW1417	0.500 µM + 0.001 µM	0.6	0.005 µM + 0.001 µM	0.12
WiDr	0.013 µM + 0.003µM	0.24	0.050 µM + 0.003µM	0.10
WiDr	0.002 µM + 0.003µM	0.18	0.008 µM + 0.003µM	0.10
LS411N	0.060 µM + 0.100µM	0.32	0.006 µM + 0.100 µM	0.07
LS411N	0.400 µM + 0.010 µM	0.45	0.030 µM + 0.050 µM	0.16

Synergistic interaction of SRCi and BRAFi across a broad range of BRAFm CRC cell lines. Representation of CI for the indicated cell lines after combined treatment with SRCi and BRAFi. Data are shown in CI format, obtained with compusyn software. CI< 0.9 means synergy. CI, combination index.

Figure S8.

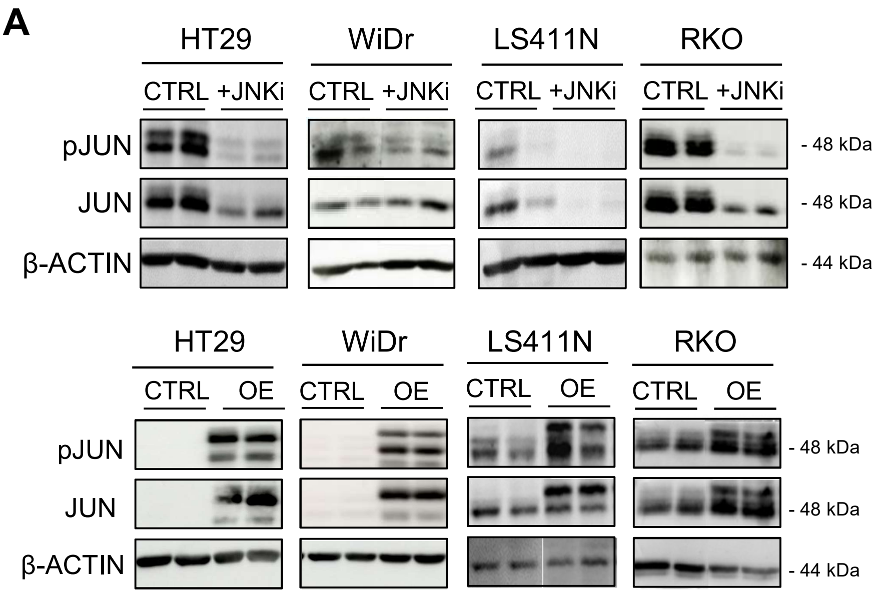

Role of c-JUN on cell survival in BRAFm CRC cell lines/ Role of JUN in cell apoptosis upon SRC and/or BRAF inhibition in BRAFm CRC cell lines. A. Representative WB for CRC cell lines after JNKi treatment and JUN overexpression are shown. β-actin levels were used as loading control. Molecular weight/size markers are indicated on the right (kDa). Images are representative of 3 replicates. **B**. Flow cytometry analysis measured by Annexin V-APC/7-AAD staining of WiDr, LS411N and RKO after treatment with JNKi (yellow color, on the left) or after c-JUN overexpression (black color, on the right); compared to control cells (in grey color), and following the indicated treatments for 24h. JNKi significantly increased apoptosis upon either single or dual SRC and/or BRAF inhibition. JUN OE decreased apoptosis upon either single or dual SRC and/or BRAF inhibition. Average and SEM of 3 independent experiments are shown. Significance was considered for * $P < 0.05$, ** $P < 0.01$, *** $P < 0.001$, and **** $P < 0.0001$. Ns, non-significant. CTRL, control. JUN, overexpression of JUN. JNKi, JNK inhibitor (30 μM). DM, DMSO. V, vemurafenib. E, encorafenib. D, dasatinib.

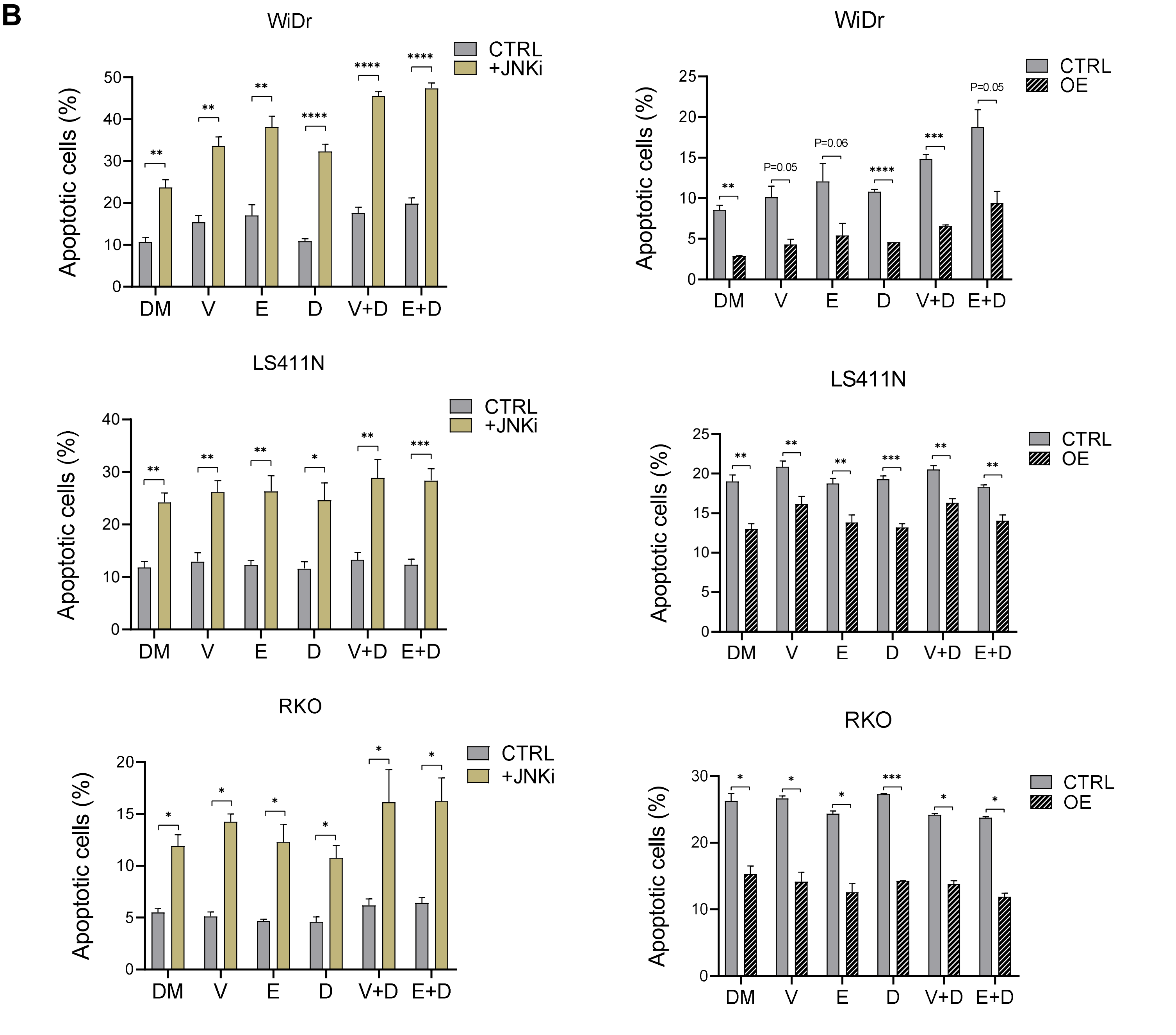

Figure S9.

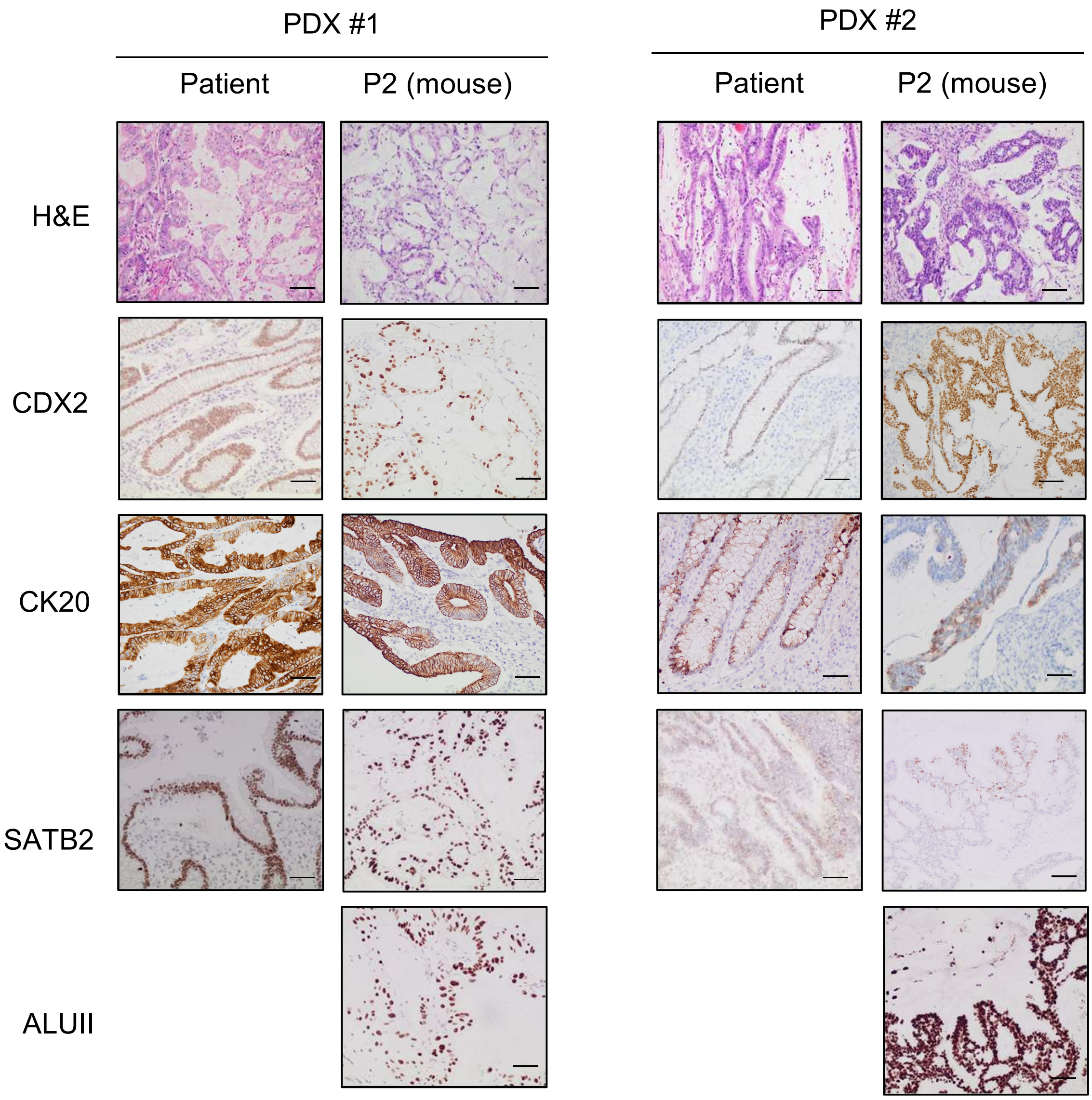

PDX characterization. A. Characterization of BRAFm CRC PDX models. Representative IHC images showing H&E, CDX2, CK20, SATB2 and ALU-II staining for PDX 1 and 2. Scale bar, 100 µm. P, passage.

Figure S10.

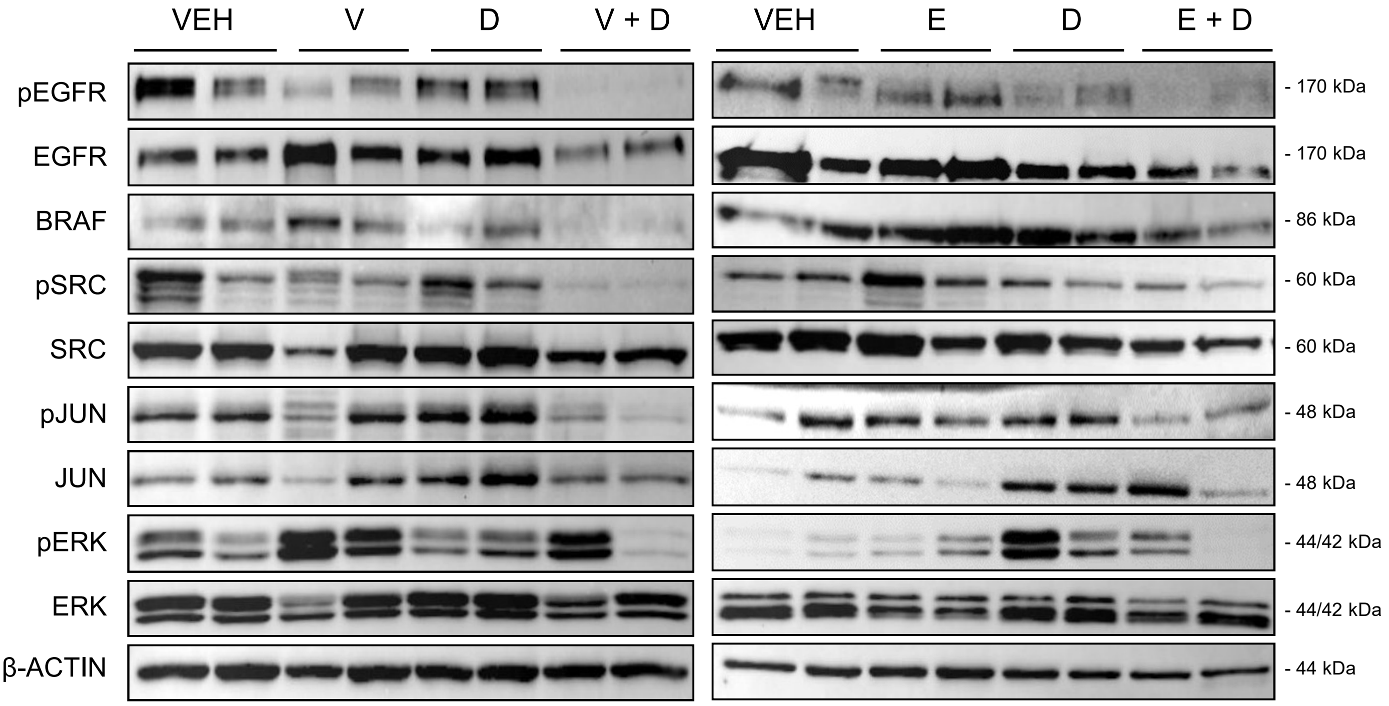

Dual inhibition of SRC and BRAF reduced the activation of the MAPK kinase pathway in CDX BRAFm CRC models. After completing treatment, mice were sacrificed and tumors from the HT29 cell line were extracted, lysed and immunoblotted with the indicated antibodies. Two representative mice were selected for each group of treatment. Components of the MAPK pathway (EGFR, BRAF, SRC, JUN and ERK) and its phosphorylated forms were evaluated. SRC activation is reflected by increased phosphorylation of the SRC activation site Y419 (pY419). β-actin was used as a loading control. Molecular weight/size markers are indicated on the right (kDa). VEH, vehicle. E, encorafenib. D, dasatinib.

Figure S11.

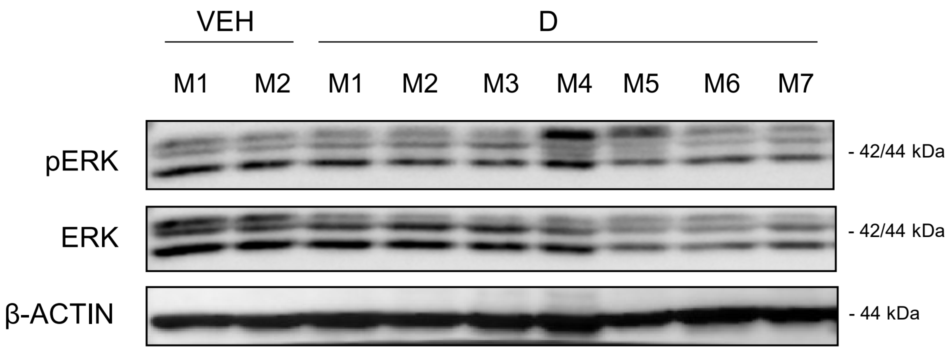

Western blot analysis of pERK, total ERK, and β-ACTIN in CDX BRAFm CRC models treated with vehicle or dasatinib (5 mg/kg). After completing treatment, mice were sacrificed and tumors from the HT29 cell line were extracted, lysed and immunoblotted with the indicated antibodies. Two representative mice were selected for vehicle and seven for dasatinib group of treatment. ERK and its phosphorylated form were evaluated. β-actin was used as a loading control. Molecular weight/size markers are indicated on the right (kDa). M, mice; VEH, vehicle; D, dasatinib.

Table S1.

Drug	Target Kinase	IC50 (nM)	Reference
**Dasatinib**	SRC	0.5	J Med Chem. 47, 2364-2377 (2004)
	Abl	<1	J Med Chem. 47, 2364-2377 (2004)
	LCK	0.4–1.1	J Med Chem. 47, 2364-2377 (2004)
	Yes	0.50	J Med Chem. 47, 2364-2377 (2004)
	C-kit	3-5	J Med Chem. 47, 2364-2377 (2004)
	PDGFRβ	28	J Med Chem. 47, 2364-2377 (2004)
	p38	100	J Med Chem. 47, 2364-2377 (2004)
	Her1	180	J Med Chem. 47, 2364-2377 (2004)
	Her2	710	J Med Chem. 47, 2364-2377 (2004)
	FGFR-1	880	J Med Chem. 47, 2364-2377 (2004)
	MEK	1700	J Med Chem. 47, 2364-2377 (2004)
**SP600125**	JNK1	0.04	Selleckchem. "SP600125." https://www.selleckchem.com/products/SP600125.html.<br>Proc Natl Acad Sci U S A. 98, 13681-13686 (2001)
	JNK2	0.04	Selleckchem. "SP600125." https://www.selleckchem.com/products/SP600125.html.<br>Proc Natl Acad Sci U S A. 98, 13681-13686 (2001)
	JNK3	0.09	Selleckchem. "SP600125." https://www.selleckchem.com/products/SP600125.html.<br>Proc Natl Acad Sci U S A. 98, 13681-13686 (2001)
	Aurora Kinase A	0.06	Selleckchem. "SP600125." https://www.selleckchem.com/products/SP600125.html.
	TRKA	0.07	Selleckchem. "SP600125." https://www.selleckchem.com/products/SP600125.html.
	FLT3	0.09	Selleckchem. "SP600125." https://www.selleckchem.com/products/SP600125.html.
	MKK4	400	Proc Natl Acad Sci U S A. 98, 13681-13686 (2001)
	MKK6	1000	Proc Natl Acad Sci U S A. 98, 13681-13686 (2001)
**Vemurafenib**	BRAF V600E	10-31	Selleckchem. "Vemurafenib (PLX4032)" https://www.selleckchem.com/products/PLX-4032.html<br>European Medicines Agency (EMA). "Zelboraf - EPAR Product Information." https://www.ema.europa.eu/en/documents/product-information/zelboraf-epar-product-information_en.pdf
	WT BRAF	10-fold over BRAF V600E	Selleckchem. "Vemurafenib (PLX4032)" https://www.selleckchem.com/products/PLX-4032.html<br>European Medicines Agency (EMA). "Zelboraf - EPAR Product Information." https://www.ema.europa.eu/en/documents/product-information/zelboraf-epar-product-information_en.pdf
**Encorafenib**	BRAF V600E	0.35	MedChemExpress. "LGX818." https://www.medchemexpress.com/LGX818.html?locale=es-ES&srsltid=AfmBOooSKSl9YmbbUUlmli7K0WEMiqKmZSE_7p1nnoCKl2V8gIiKOfmJ<br>European Medicines Agency (EMA). "Braftovi - EPAR Product Information." https://www.ema.europa.eu/en/documents/product-information/braftovi-epar-product-information_en.pdf
	WT BRAF	0.47	European Medicines Agency (EMA). "Braftovi - EPAR Product Information." https://www.ema.europa.eu/en/documents/product-information/braftovi-epar-product-information_en.pdf
	CRAF	0.30	European Medicines Agency (EMA). "Braftovi - EPAR Product Information." https://www.ema.europa.eu/en/documents/product-information/braftovi-epar-product-information_en.pdf

***In vitro* IC50 values of dasatinib, SP600125, vemurafenib and encorafenib against their main target kinases.** IC50 values indicate the half-maximal inhibitory concentration. The data highlight the specificity and potency of Dasatinib (TKi), SP600125 (JNKi), vemurafenib and encorafenib (BRAFi) against various kinases, including SRC family kinases, Abl, and MAPK/JNK cascade kinases. WT, wild-type.

Table S2.

Antibodies	Company	Reference	Application (Dilution)
**Primary**			
**c-JUN**	Cell Signaling	9165	WB (1:700)
**EGFR**	Cell Signaling	4267	WB (1:1000)
**p44/42 MAPK (Erk1/2)**	Cell Signaling	4695	WB (1:1000)
**Phospho-c-JUN (Ser73)**	Cell Signaling	9164	WB (1:500)
**Phospho-EGFR (Tyr1068)**	Cell Signaling	2234	WB (1:1000)
**Phospho-p44/42 MAPK (Erk1/2) (Thr202/Tyr204)**	Cell Signaling	9101	WB (1:1000)
**Phospho-SAPK/JNK (Thr183/Tyr185)**	Cell Signaling	9251	WB (1:700)
**Phospho-SRC (Tyr 416)**	Cell Signaling	6943	WB (1:700)
**SAP/JNK**	Cell Signaling	9252	WB (1:1000)
**SRC**	Abcam	ab109381	WB (1:1000)
**β-actin**	Cell Signaling	12262	WB (1:5000)
**ALU II**	Roche	05272041001	IHC (prediluted)
**CDX2**	Dako	IR080	IHC (prediluted)
**CDX2**	Dako	IR080	IHC (prediluted)
**CK20**	Dako	IR777	IHC (ready to use)
**SATB2**	Cell marque	384R-15	IHC (1:200)
**MLH1 (G168-728)**	Leica	PA0988	IHC
**MSH2 (G219-1129)**	Leica	PA0989	IHC
**MSH6 (Clone 44)**	Leica	PA0990	IHC
**PMS2 (A-16-2)**	Leica	PA0991	IHC
**Secondary**			
Anti-mouse IgG	Cell Signaling	7076	WB (1:10000)
Anti-rabbit-IgG	Cell Signaling	7074	WB (1:10000)

Antibodies used in this study. IHC, Immunohistochemistry. WB, western blot.

Table S3.

	Drug concentration (µM)					
**Assay**	**Drug**	**HT29**	**SW1417**	**WiDr**	**LS411N**	**RKO**
**Migration**	**Dasatinib**	0.100	0.001	0.003	-	0.100
	**Vemurafenib**	0.400	0.080	0.080	-	1.850
	**Encorafenib**	0.080	0.005	0.008	-	0.016
**CFA**	**Dasatinib**	0.100	0.001	0.003	-	0.100
	**Vemurafenib**	0.140	0.080	0.080	-	0.600
	**Encorafenib**	0.080	0.030	0.050	-	0.016
**Apoptosis**	**Dasatinib**	0.100	-	0.030	0.100	0.100
	**Vemurafenib**	0.400	-	1.500	0.350	1.850
	**Encorafenib**	0.080	-	2.000	0.001	2.000
**Cell cycle**	**Dasatinib**	0.100	-	0.020	0.100	0.100
	**Vemurafenib**	0.400	-	0.100	2.100	1.850
	**Encorafenib**	0.080	-	0.300	0.370	0.600

Drug concentrations used in *in vitro* assays. CFA, cell colony formation.

Supplementary Materials and Methods

Cell lines, cell culture and genetic alterations

HT29 were cultured in McCoy 5-A medium (Gibco, Fisher Scientific, Madrid, Spain) supplemented with 10% fetal bovine serum (FBS, TICO Europe, FBSEU500) and 1% antibiotic antimycotic solution (AAS) (penicillin-streptomycin-amphotericin, ref: A5955, Merck, Madrid, Spain). SW1417, 293T and A375 cells were grown in Dulbecco's modified Eagle medium (DMEM; Merck) with 10% FBS, and 1% AAS. LS411N and WiDr cells were cultured in RPMI-1640 medium (Merk) with 10% FBS and 1% AAS, and RKO cells were cultured in Eagle's Minimum Essential medium (EMEM, Merck) with 15% FBS and 1% AAS. Cell lines were authenticated by short-tandem repeat profiling in the CNIO genomic service (WiDr: 02/06/2021. RKO, LS411N: 24/03/2023. HT29:06/06/2023), and tested negative for mycoplasma contamination.

Knock-out and overexpression of SRC in human colorectal cancer cell lines

KO cells were generated by using a CRISPR/Cas single guide (sg)RNA targeting SRC (F: CACCGATAGTCATAGAGGGCCACAA; R: TGTGGCCCTCTATGACTATC), which was designed using the Broad Institute web tool (https://www.broadinstitute.org/rnai/public/analysis-tools/sgrna-design). The sgRNA was cloned into the lentiCRISPR v2 (Addgene, 52961, Watertown, USA). Empty vector (non-targeting vector) was used as a negative control. For the overexpression (OE), the lentiORF clone of Human v-src sarcoma (Schmidt-Ruppin A-2) viral oncogene homolog (avian) (SRC) (RC208622L4) was used (Quimigen, Madrid, Spain). pGIPz-GFP-EV (Addgene, 120933) was used as a negative control. KO and OE cells, packaging vectors (PMD2G (Addgene, 12259), pCD/NL-BH*DDD (Addgene, 17531)) and sgRNAlentiCRISPR or SRC lentiORF plasmids were transfected into 293T cells. For this, 293T cells (60% confluent) were seeded in 10 cm-diameter dishes and plasmids were co-transfected using Lipofectamine 2000 reagent (Thermo Fisher Scientific, Madrid, Spain) according to the manufacturer's instructions. 293T medium containing virus was collected 48 h post-transfection to infect target cells in 10 cm-diameter dishes (60% confluent) at 37˚C. Post-infected cells were treated with 4 µg/mL puromycin (Merck) at 37˚C for 3 weeks to select the cell clones with stable SRC modification for further studies. Single clones were used to perform functional assays.

CRC cell line xenograft and PDX models

In CDX and PDX models, when tumor volume reached 150-200 mm^3, mice were randomized with rolling enrollment into vehicle or different treatment groups: vehicle (5 % DMSO, 40% PEG300, 5% Tween80 and 50% H20; or 4 % DMSO, 30% PEG300, 5% Tween80 and 61% H20), cetuximab (20 mg/kg), vemurafenib (0.63 mg/kg), encorafenib (3 mg/kg), dasatinib (5 mg/kg) or their combination. More details about drug solvents can be found in Drugs section. The sample size was determined based on the significance level, desired power, and expected variability to ensure reliable results. All treatments, except for cetuximab, were intraperitoneally administered five days a week for a total of 20 doses. Cetuximab was intraperitoneally administered twice a week for a total of 8 doses. Tumor volume was evaluated three times a week by digital calipers and calculated by the following formula: (small diameter)^2 x large diameter x 0.52. The maximum tumor size permitted was not exceeded. All mice were monitored for signs of toxicity (for example, weight loss). Mice were weighed twice a week. After 20 doses of treatment, mice were euthanized by CO_2 inhalation and tumors were collected for further analysis.

Details about patients:

-Patient #1:86-yead-old male, mucinous adenocarcinoma of the right colon, G2 pT3N1M0, stage IIIB at diagnosis, K-N-RAS WT, BRAF V600E, dMMR (IHQ: MLH1/PMS2 negative).

-Patient #2: 83-yead-old female, mucinous adenocarcinoma of the right colon, GX pT4aN2aM0, stage IIIB at diagnosis, K-RAS WT, NRAS A146T-V, BRAF V600E, dMMR (IHQ: MLH1/PMS2 negative).''

Histopathology and molecular analysis

Harvested tissues from all PDX models were fixed in 10% buffered formalin within 30 min of resection. After 24 h of tissue fixation, standard procedures were followed for further tissue processing. Hematoxylin and eosin (H&E) and immunohistochemistry (IHC) were performed by staining tissue sections with antibodies against CDX2, CK20, ALU II and SAT-B2 (Supplementary Table 1), and independently evaluated by two experienced pathologist in our institution.

IHC staining for MLH1, PMS2, MSH2, and MSH6 was performed on formalin-fixed, paraffin-embedded (FFPE) tumor tissue blocks by standard BOND-MAX automated staining methods. For the assessment of MSI status by PCR-based fragment-sizing test, five microsatellite mononucleotide loci, namely BAT25, BAT26, NR21, NR24, and MONO27, were analyzed using OncoMate MSI Dx Analysis System according to the manufacturer's protocol (Promega Corporation, Madrid, Spain). Briefly, the 5 microsatellite markers were amplified using a multiplex fluorescence PCR and subjected to capillary electrophoresis on SeqStudio Genetic Analyzer (Thermo Fisher Scientific, Madrid, Spain). MSI was scored when at least 2 of 5 loci were unstable. Details on the antibodies used are provided in Supplementary Table 1.
